# Rhodiola rosea-derived exosome-like nanovesicles inhibit vascular endothelial pyroptosis in the treatment of limb skeletal muscle ischemic injury through the TXNIP/NLNP3 pathway

**DOI:** 10.1093/rb/rbaf113

**Published:** 2025-10-31

**Authors:** Dachang Liu, Zhibin Zhang, Yuchao Wang, Xiaoyu Liang, Yun Chang, Changduo Wang, Yaming Guo, Shijie Zhang, Jianghui Zhou, Meng Zhang, Hechen Shen, Xuesong Zhang, Wenqing Gao

**Affiliations:** School of Medicine, Nankai University, Tianjin 300071, China; Tianjin Key Laboratory of Extracorporeal Life Support for Critical Diseases, Tianjin University Central Hospital; Department of Heart Center, The Third Central Hospital of Tianjin; Nankai University Affiliated Third Center Hospital; The Third Central Clinical College of Tianjin Medical University, Tianjin 300170, China; Senior Department of Orthopedics, The Fourth Medical Centre, Chinese PLA Hospital, Beijing 100048, China; School of Medicine, Nankai University, Tianjin 300071, China; Tianjin Key Laboratory of Extracorporeal Life Support for Critical Diseases, Tianjin University Central Hospital; Department of Heart Center, The Third Central Hospital of Tianjin; Nankai University Affiliated Third Center Hospital; The Third Central Clinical College of Tianjin Medical University, Tianjin 300170, China; School of Medicine, Nankai University, Tianjin 300071, China; Tianjin Key Laboratory of Extracorporeal Life Support for Critical Diseases, Tianjin University Central Hospital; Department of Heart Center, The Third Central Hospital of Tianjin; Nankai University Affiliated Third Center Hospital; The Third Central Clinical College of Tianjin Medical University, Tianjin 300170, China; School of Medicine, Nankai University, Tianjin 300071, China; Tianjin Key Laboratory of Extracorporeal Life Support for Critical Diseases, Tianjin University Central Hospital; Department of Heart Center, The Third Central Hospital of Tianjin; Nankai University Affiliated Third Center Hospital; The Third Central Clinical College of Tianjin Medical University, Tianjin 300170, China; Tianjin ECMO Treatment and Training Base, Tianjin 300170, China; School of Medicine, Nankai University, Tianjin 300071, China; Tianjin Key Laboratory of Extracorporeal Life Support for Critical Diseases, Tianjin University Central Hospital; Department of Heart Center, The Third Central Hospital of Tianjin; Nankai University Affiliated Third Center Hospital; The Third Central Clinical College of Tianjin Medical University, Tianjin 300170, China; Tianjin ECMO Treatment and Training Base, Tianjin 300170, China; State Key Laboratory of Advanced Medical Materials and Devices, Institute of Biomedical Engineering, Chinese Academy of Medical Science & Peking Union Medical College, Tianjin 300192, China; State Key Laboratory of Advanced Medical Materials and Devices, Institute of Biomedical Engineering, Chinese Academy of Medical Science & Peking Union Medical College, Tianjin 300192, China; School of Medicine, Nankai University, Tianjin 300071, China; Tianjin Key Laboratory of Extracorporeal Life Support for Critical Diseases, Tianjin University Central Hospital; Department of Heart Center, The Third Central Hospital of Tianjin; Nankai University Affiliated Third Center Hospital; The Third Central Clinical College of Tianjin Medical University, Tianjin 300170, China; Tianjin Key Laboratory of Extracorporeal Life Support for Critical Diseases, Tianjin University Central Hospital; Department of Heart Center, The Third Central Hospital of Tianjin; Nankai University Affiliated Third Center Hospital; The Third Central Clinical College of Tianjin Medical University, Tianjin 300170, China; Tianjin Key Laboratory of Extracorporeal Life Support for Critical Diseases, Tianjin University Central Hospital; Department of Heart Center, The Third Central Hospital of Tianjin; Nankai University Affiliated Third Center Hospital; The Third Central Clinical College of Tianjin Medical University, Tianjin 300170, China; Department of Thoracic Surgery, Sichuan Provincial People’s Hospital, School of Medicine, University of Electronic Science and Technology of China, Chengdu 610000, China; School of Medicine, Nankai University, Tianjin 300071, China; Senior Department of Orthopedics, The Fourth Medical Centre, Chinese PLA Hospital, Beijing 100048, China; School of Medicine, Nankai University, Tianjin 300071, China; Tianjin Key Laboratory of Extracorporeal Life Support for Critical Diseases, Tianjin University Central Hospital; Department of Heart Center, The Third Central Hospital of Tianjin; Nankai University Affiliated Third Center Hospital; The Third Central Clinical College of Tianjin Medical University, Tianjin 300170, China; Tianjin ECMO Treatment and Training Base, Tianjin 300170, China

**Keywords:** RhELNs, skeletal muscle, vascular

## Abstract

Skeletal muscle ischemia, resulting from impaired blood flow, is a prevalent clinical issue and a leading cause of amputation. Plant-derived exosome-like nanovesicles (ELNs) have emerged as promising candidates due to their diverse bioactive components with antioxidant, anti-inflammatory and regenerative properties. In this study, exosome-like nanovesicles (RhELNs) extracted from the root of Rhodiola rosea, a traditional Chinese medicine, were proved to have a good ability to promote the regeneration of vascular endothelial cells under hypoxia by EdU experiment and transwell experiment *in vitro*. After the treatment of mice with RhELNs injected into the tail vein, it was found that the inflammation level of the skeletal muscle of the mice was decreased, the degree of fibrosis was alleviated, the blood flow was restored, skeletal muscle atrophy and limb gangrene were improved. These results indicate that RhELNs can promote the recovery of ischemic skeletal muscle and angiogenesis. Furthermore, we identified a novel microRNA (novel-mirNA-115-5p) in RhELNs, which plays an important role in RhELNs. It protects vascular endothelial cells from mitochondrial damage by targeting TXNIP and it promotes vascular regeneration by reducing cellular pyroptosis under hypoxia conditions through inhibition of the TXNIP- NLRP3 pathway. These results suggest that RhELNs represent a promising new approach for treating lower limb skeletal muscle ischemic diseases.

## Graphical abstract

**Figure:**
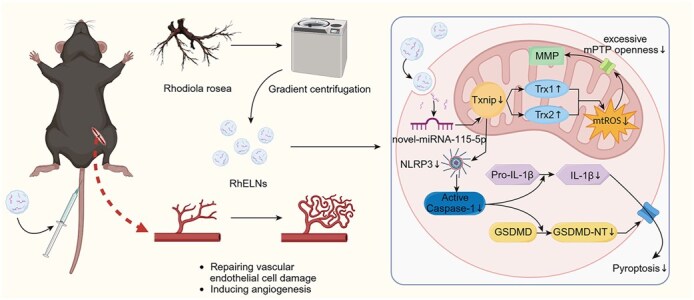

## Introduction

Critical limb ischemia (CLI), frequently associated with diabetes, trauma, and severe arterial disease, can result in muscle atrophy, ischemic ulcers, non-healing wounds, and foot gangrene, potentially progressing to severe outcomes like amputation and death if not adequately managed [1–3]. The primary pathological features of CLI are ischemia- and hypoxia-induced cell death and impaired angiogenesis, with vascular endothelial cells (ECs), which form the innermost layer of blood vessels and are highly vulnerable to ischemic and hypoxic conditions, playing a pivotal role in the disease’s progression [4, 5]. Therefore, protecting ECs from ischemia- and hypoxia-induced damage is critical for effective CLI treatment. In recent years, extracellular vesicles have emerged as a novel therapeutic strategy for lower limb ischemia, alongside traditional approaches being evaluated in clinical trials.

Over the past decade, interest in plant-derived exosome-like nanovesicles (ELNs) has surged due to their bioactive composition, including lipids, proteins, and nucleic acids that facilitate intercellular signaling. Compared with exosomes derived from animal sources, their widespread availability, superior biocompatibility, low immunogenicity, cost-effectiveness, and ease of accessibility make them promising candidates for further exploration in the treatment of CLI [6–9]. Additionally, compared to conventional drugs, ELNs contain multiple bioactive components and exhibit inflammation-targeting properties, facilitating their accumulation at sites of inflammation.

Rhodiola rosea, an ancient wild herb native to high-altitude rocky regions, has a long history of use in traditional medicine across Asia, Europe, and the Americas due to its broad habitat range [10]. Known for its diverse pharmacological effects, including anti-hypoxia, anti-aging, anti-inflammatory, antioxidant, and cardiovascular protective properties, Rhodiola rosea has been widely studied [10–12]. However, research on exosome-like nanovesicles derived from fresh Rhodiola rosea (RhELNs) remains extremely limited, making the investigation of RhELNs for the treatment of lower limb ischemia a promising area of study. Plant-derived exosome-like nanovesicles, including RhELNs, contain various microRNAs (miRNAs) that mediate cell-to-cell interactions, playing significant roles in inflammation, tissue repair, and fibrosis [13, 14].

Thioredoxin-interacting protein (TXNIP) is a multifunctional protein critical for regulating cellular homeostasis and redox balance. It is activated by various cellular stresses, such as inflammation and oxidative stress, and facilitates the release of inflammatory cytokines [15]. TXNIP inhibits thioredoxins 1 and 2 (TRX1 and TRX2), promoting the excessive generation of mitochondrial reactive oxygen species (mtROS) and oxidative stress, leading to apoptosis *via* mitochondrial damage [16, 17]. Moreover, the dissociation of the TRX-TXNIP complex enables TXNIP to interact with the Nod-like Receptor Protein 3 (NLRP3) inflammasome, further driving inflammation [18]. Activation of NLRP3 subsequently triggers Caspase-1, cleaving gasdermin D (GSDMD), which induces vascular endothelial cell death and worsens the prognosis of lower limb ischemia [19, 20]. Thus, targeting endothelial TXNIP is essential for enhancing post-ischemic blood flow recovery [21].We found that RhELNs could inhibit the expression of TXNIP in human aortic endothelial cells (HAECs) under hypoxia and could reduce the production of ROS in HAECs under hypoxia. We speculated that RhELNs possibly reduced ROS production by inhibiting TXNIP expression through their contained miRNAs, so we performed high-throughput sequencing of RhELNs and found a novel miRNA, novel-miRNA-115-5p, which was predicted to be well-targeted to TXNIP by miRanda software analysis. This study aims to investigate the potential of RhELNs to inhibit NLRP3 activation through the novel-miRNA-115-5p-TXNIP pathway, thereby alleviating vascular endothelial cell death in the treatment of lower limb ischemia.

## Materials and methods

### Preparation and characterization of Rhodiola rosea source exudate

Fresh Rhodiola rosea rhizomes from China were processed by washing, sterilization, and juicing in phosphate-buffered saline (PBS) (Beyotime, China) to prepare the stock solution. The extract was subjected to a series of centrifugation steps to isolate ELNs: initial centrifugation at 3000 g for 30 min to remove cellular components, followed by 10 000 g for 60 min to clear additional debris, and 150 000 g for 90 min to concentrate the ELNs. To further purify and concentrate the ELNs, gradient centrifugation was conducted using sucrose solutions of 8%, 30%, 45% and 60%, with an additional 150 000 g centrifugation for 60 min. The ELNs collected from the sucrose gradient interface underwent a final purification step with 150 000 g centrifugation in PBS for 60 min, and sterilization was achieved by filtration through a 0.22 μm membrane. The purified ELNs were characterized by transmission electron microscopy (HITACHI, Japan) to observe their morphology, and their size distribution was measured using a nanoparticle analyzer (PARTICLE METRIX, Germany). The zeta potential was also assessed using a zeta potential analyzer to evaluate surface charge properties.

### Component analysis of RhELNs

We added a certain amount of methanol to the extracted homogenized solution sample of RhELNs for metabolite extraction. The mixture was sonicated and centrifuged at 4°C for 20 min. 20 μL of the supernatant of each sample was taken into the injection bottle for machine detection, and the samples were analyzed by mass spectrometry using the ultra performance liquid chromatography system and mass spectrometer.

### Cell culture and treatment

HAECs were cultured in Dulbecco's Modified Eagle Medium (DMEM) medium (Gibco, USA) supplemented with 10% fetal bovine serum, penicillin, and streptomycin at 37°C and 5% CO_2_, obtained from Peking Union Medical College. By adjusting the gas environment (37°C, 5% carbon dioxide and 1% oxygen) and restricting serum in the culture medium to simulate hypoxic conditions, to mimic physiological ischemic hypoxia.

### Effects of RhELNs on vascular endothelial cell viability

To evaluate the impact of RhELNs on HAEC viability, cells were seeded in 96-well plates and incubated under normoxic and hypoxic conditions for 24 h. During the hypoxic exposure, RhELNs were administered at protein concentrations ranging from 10 to 100 μg/mL. Untreated cells served as controls. Cell viability was assessed using a CCK-8 assay. For further analysis, two concentrations were selected: 50 μg/mL (designated as Hypoxia+RhELNs (L)) and 100 μg/mL (Hypoxia+RhELNs (H)) to represent low and high dosage groups, respectively.

### Exosome cell uptake

For RhELN uptake studies, HAECs were cultured overnight in 24-well plates containing small round glass coverslips. RhELNs were fluorescently labeled with DiR (Beijing Bioss Biotechnology Co., Ltd, China) and then added to the cultures. Cells were co-incubated with the labeled RhELNs for 2, 4 and 8 h. After each incubation period, cultures were aspirated and washed with PBS to remove unbound vesicles. Cells were then fixed in 4% paraformaldehyde (Beijing Solarbio Science & Technology Co., Ltd, China), and the coverslips were mounted with 4',6-diamidino-2-phenylindole (DAPI) Fluoromount-G (SouthernBiotech, USA) for nuclear counterstaining. The uptake of RhELNs by HAECs was visualized using a confocal microscope (ZEISS, Germany).

### *In vitro* migration of HAECs

The effect of RhELNs on the migratory ability of HAECs was evaluated using both Transwell and cell scratch assays.

For the transwell assay, HAECs in Petri dishes were digested, centrifuged, and then resuspended in DMEM. We added 500 μL of DMEM medium containing more FBS to each well of a 24-well plate. Transwell inserts were placed in the well plates. Then, 200 μL of DMEM containing 1 × 10^4^ HAECs was added to the inserts. These cells were cultured under different conditions for 24 h. Next, the medium in inserts was aspirated out. Then, the inserts were cleaned with PBS and placed in 4% paraformaldehyde for fixation. After the inserts were washed with PBS, we carefully removed the non-migrated cells from the transwell inserts. After that, the migrated cells on the inserts were stained with 0.1% crystal violet stain for 20 min. The cells that had migrated through the membrane were visualized and photographed using a microscope.

For the scratch assay, we created scratches through a 200 μL pipette on the plane of the well plate full of cells. The cells were then incubated under different conditions for 24 h. After the incubation period, the plates were photographed again and the results of the cell scratching experiment were analyzed using ImageJ software.

### EdU assay for cell proliferation ability

The proliferative capacity of HAECs was assessed using the BeyoClick^TM^ EdU cell proliferation kit with Alexa Fluor 488 (Beyotime, China). HAECs were seeded in 96-well plates and incubated overnight. Following a change in medium and the administration of treatments, 20 μL of EdU was added and the cells were incubated at 37°C for 2 h. The cells were then fixed with 4% paraformaldehyde, permeabilized with 0.3% TritonX-100, and stained with Hoechst. Fluorescent imaging was performed, and the results were analyzed with ImageJ.

### Angiogenesis assay

For the angiogenesis assay: Growth factor-reduced matrix gel (Corning, USA) was incubated overnight at 4°C. The gel was then aliquoted into 24-well plates, spread evenly, and incubated at 37°C for 1 h. HAECs (3 × 10^4^ cells) were resuspended in DMEM and seeded onto the matrix gel surface. Each condition was tested in triplicate. After an 8-h incubation at 37°C, the formation of tubular structures was imaged, and data were quantified using ImageJ.

### TUNEL staining

The inhibitory effect of RhELNs on HAEC apoptosis was evaluated through TUNEL staining, conducted with TUNEL reagent (Beyotime, China). Fluorescence microscopy was used for imaging.

### Lower limb ischemia model and animal experimental treatment methods

C57BL/6J mice (6–8 weeks old) obtained from Beijing Vital River Laboratory Animal Technology Co. were anesthetized with 2.5% isoflurane and positioned in dorsal recumbency with externally rotated hind limbs. A skin incision above the femoral artery was made from the inguinal ligament, followed by double ligation using sutures. Subcutaneous blood flow in the lower extremities was assessed *via* laser Doppler imaging (Perimed Instruments) at baseline, 7 days, 14 days and 21 days post-surgery. Mice were euthanized at 7 and 14 days. All animal procedures were ethically reviewed and approved by the Experimental Animal Ethics Committees of the Institute of Radiation Medicine, Chinese Academy of Medical Sciences and Peking Union Medical College (Approval No. IRM/2-IACUC-2410-011). Mice were treated with RhELNs at a dose of 10 mg/kg *via* intravenous injection, which was administered every two days.

### Skeletal muscle hematoxylin and eosin staining

Muscle tissues were sequentially dehydrated by placing them in different concentrations of alcohol solutions. The dehydrated skeletal muscle tissues were embedded in paraffin. We paraffin fast cut the skeletal muscle tissues into 2 μm thick sections. Afterwards, we stained the paraffin sections of skeletal muscle tissue using hematoxylin and eosin staining kit (Beijing Solarbio Science & Technology Co., Ltd, China) according to the instructions.

### MASSON staining of skeletal muscle

Masson trichrome staining was applied to paraffin-embedded muscle sections using the Masson staining kit (Beijing Solarbio Science & Technology Co., Ltd, China) for fibrosis assessment.

### Tissue immunofluorescence staining

After deparaffinization and hydration, antigen retrieval was conducted by heating the sections in 0.01 mol/l sodium citrate buffer (pH 6.0) using a microwave. The sections were then incubated overnight at 4°C with primary antibodies: CD31 (Abcam, 1:50) and α-SMA (Proteintech, 1:200). After washing, the sections were incubated with secondary antibodies conjugated to Alexa Fluor 488 or Alexa Fluor 594 (Affinity, China). Finally, cell nuclei were counterstained with DAPI. In addition, we quantified the fluorescence intensity of each immunofluorescence picture by ImageJ software, and the results of the fluorescence intensity of the different groups/the fluorescence intensity of the control group were used as the quantification results of the relative fluorescence intensity.

### Enzyme-linked immunosorbent assay

On the 7th day of the experiment, skeletal muscle tissues were collected from mice, washed with PBS to remove residual blood, weighed and cut into pieces. Then, PBS was added to the cut tissues and the skeletal muscle tissues were thoroughly ground using a tissue grinder. After that, the obtained tissue homogenate was centrifuged at 5000 ×g for 5 min, and the supernatant was collected for detection. The levels of TNF-α and IL-6 were detected by enzyme-linked immunosorbent assay (ELISA) kit (Beijing Solarbio Technology Co., LTD., China). In addition, we injected RhELNs into mice, and on the 14th day, we collected blood samples from the mice, centrifuged the blood samples at 2000×g for 10 min to separate the serum, and detected the levels of IL-1β, CRP and IL-8 in the serum of the mice using ELISA kits.

### Laser Doppler flow imaging

Blood flow in the lower limbs was assessed using a Laser Doppler flow imager (Moor, UK) on days 0, 7, 14 and 21, with the perfusion ratio between the treated and contralateral limbs calculated for each treatment group.

### Limb ischemia score

The evaluation method is as follows: 0 = normal, 1 = discoloration of one nail, 2 = discoloration of more than two nails, 3 = partial discoloration of one toe, 4 = partial discoloration of more than two toes, 5 = complete discoloration of one toe, 6 = complete discoloration of more than two toes, 7 = foot necrosis, 8 = Extensive foot necrosis.

### Organ toxicity testing

At day 14, the heart, liver, spleen, lung and kidney were collected from both exosome-treated and untreated control mice. Hematoxylin and eosin (HE) staining was used to evaluate the tissue morphology for any pathological changes.

### Dual-luciferase reporter gene test

Sangon Biotech (Shanghai) Co., Ltd synthesized sequences corresponding to the 3′ UTR of TXNIP mRNA, including wild-type (WT) and mutant (MUT) versions of the novel-miRNA-115-5p binding site. These sequences were incorporated into dual-luciferase reporter plasmids, which were co-transfected into cells. Luciferase activity was measured 48 h post-transfection using the Dual-Luciferase Reporter Gene Assay System to determine the effect of novel-miRNA-115-5p on TXNIP expression.

### qPCR analysis

After co-culturing RhELNs with HAECs, total RNA was collected and reverse transcribed using the total RNA extraction kit (Beijing Solarbio Technology Co., LTD., China). After synthesizing the amplified cDNA, we performed qPCR with SYBR Green Master Mix kit and detected the relative expression of novel-miRNA-115-5p in cells. The primer of novel-miRNA-115-5p was: ACGGCACGCTTGATCGTGAC (FORWARD); ATCCAGTGCAGGGTCCGAGG (REVERSE).

### Western blot

Samples were resolved by SDS-PAGE before being transferred onto PVDF membranes (Bio-Rad Laboratories, USA). The membranes were blocked with a blocking solution (Beyotime, China). Primary antibodies were applied at 4°C overnight, and after another three washes, secondary antibodies were added. Protein signals were detected using an ECL chemiluminescence kit (Beyotime, China), with images captured and quantified using ImageJ software. The primary antibodies included anti-TXNIP (Proteintech, China), anti-NLRP3 (Proteintech, China), anti- cleaved caspase-1 (Cell Signaling Technology, USA), anti-GSDMD-NT (Affinity, China), anti-IL-1β (Abcam, USA) and anti-β-actin (Abcam, USA).

### Transfection

Novel-miRNA-115-5p mimics, inhibitors, and corresponding negative controls (Mimics NC and Inhibitor NC) were obtained from Sangon Biotech Co., Ltd (Shanghai). Plasmids for overexpression were also sourced from the same company, and assays were performed 48 h post-transfection.

### Mitochondrial membrane potential

We detected the mitochondrial membrane potential (ΔΨm) of HAECs using JC-1 staining solution (Beyotime, China).

### Reactive oxygen species monitoring

Cellular ROS and mitochondrial ROS production were quantified using the reactive oxygen species assay kit (Beyotime, China) and mitochondrial superoxide detection kit (Beyotime, China).

### Cellular immunofluorescence

Cells were fixed by 4% paraformaldehyde solution for 15 min. We permeabilized the cells with 0.2% Triton X-100. Next the cells were placed in PBS solution containing 5% bovine serum albumin and incubated for 30 min. After three PBS washes, primary antibodies were added and the cells were incubated for more than 12 h at 4°C. After washing the cells with PBS, we added the secondary antibody to the cells and incubated them for 1 h, after which we stained the nuclear with DAPI. Finally, the cell samples were photographed by fluorescence microscope.

### Mitochondrial permeability transition pore assay

Vascular endothelial cells were cultured in well plates and washed with PBS. Then, the mPTP opening of each group of vascular endothelial cells was detected using the mPTP fluorescence assay kit (Beyotime, China) according to the manufacturer’s instructions. Next, we stained the nuclei. Finally, we photographed them by fluorescence microscopy.

### Data statistical analysis methods

We used GraphPad Prism software for statistical analysis of data, two independent samples t-test for comparison between two groups, and one-way ANOVA analysis for multiple group comparisons of three or more groups under the condition of satisfying homogeneity of variance. *P* < 0.05 was considered statistically significant. * represents *P* < 0.05, ** represents *P* < 0.01,*** represents *P* < 0.001, and ns represents no statistically significant difference.

## Results

### Extraction and characterization of RhELNs

**Figure 1.**
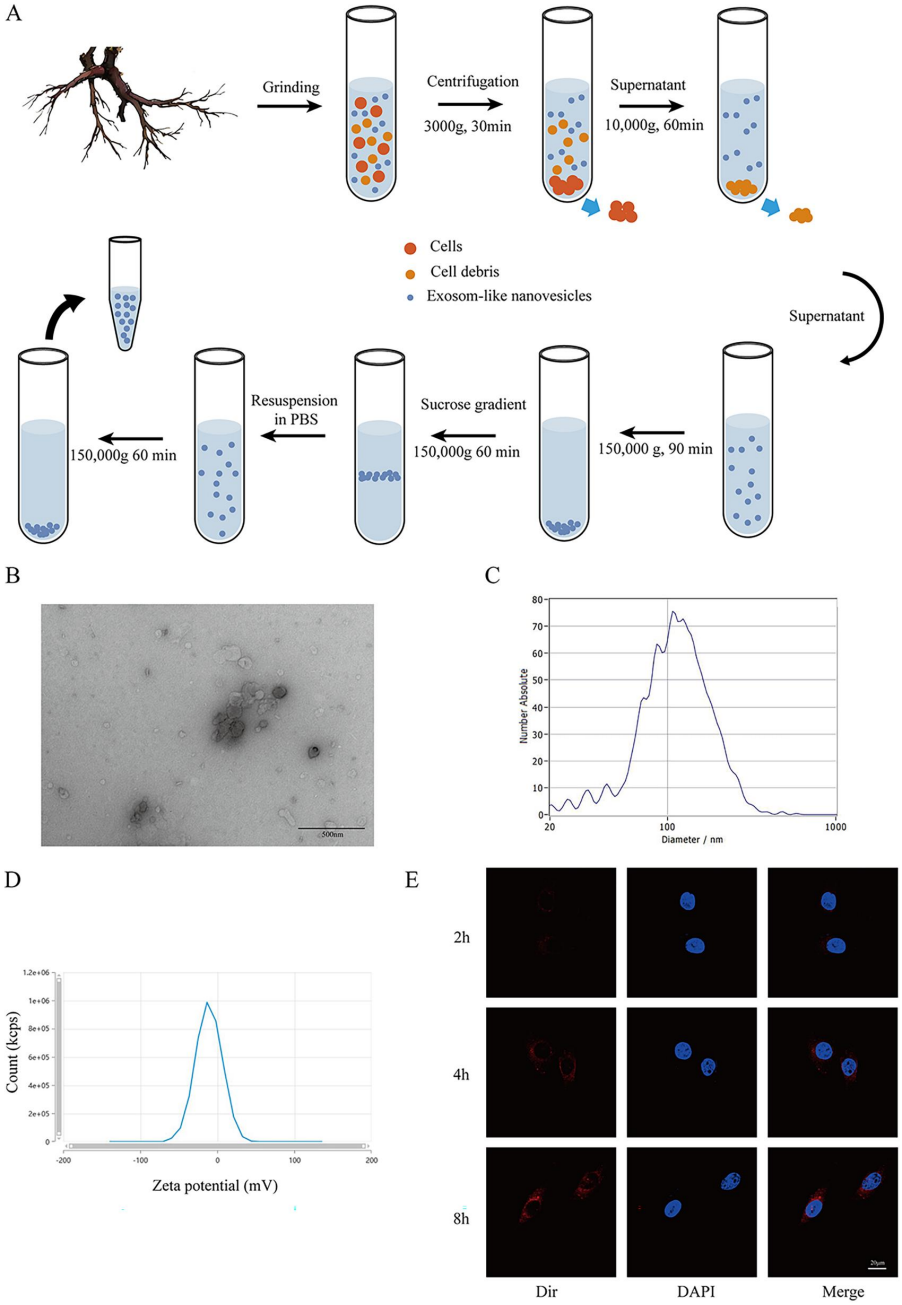
Extraction and characterization of RhELNs. (**A**) Schematic overview of RhELN extraction from Rhodiola rosea. (**B**) Transmission electron microscopy images showing RhELNs morphology. (**C**) Particle size distribution of RhELNs. (**D**) Zeta potential analysis of RhELNs. (**E**) Cellular uptake of RhELNs by HAEC observed under confocal microscopy.

RhELNs were successfully extracted (Figure 1A). Their morphology and size distribution were characterized using transmission electron microscopy (TEM) and nanoparticle tracking analysis, revealing a particle size range of 30–200 nm, consistent with exosome criteria (Figure 1B and C). The average zeta potential measured at −11.1 mV indicated good colloidal stability (Figure 1D). Confocal microscopy showed progressive uptake of RhELNs by HAECs (Figure 1E). In addition, we performed metabolite analysis of RhELNs, which primary metabolites mainly included substances such as lipids, organic acids, amino acids and derivatives carbohydrates. Its secondary metabolites mainly included substances such as phenols and derivatives, flavonoids, alkaloids, terpenoids, etc. (Supplementary Figures S5 and S6).

### RhELNs can promote HAEC repair under hypoxia

**Figure 2.**
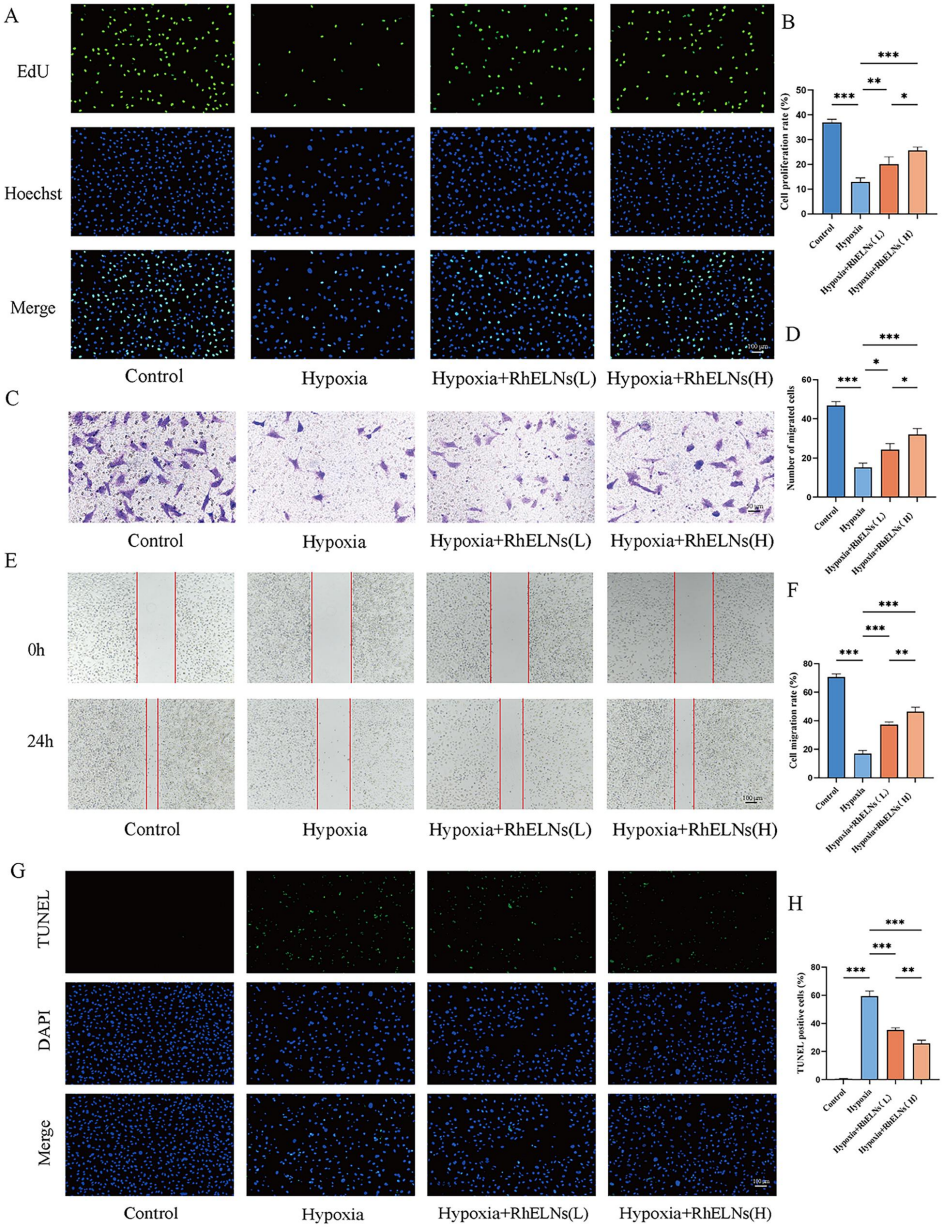
Effects of RhELNs on HAEC repair under hypoxia. (**A**) EdU assay results showing the impact of RhELNs on HAEC proliferation under hypoxic conditions. (**B**) Quantitative analysis of EdU assay results. (**C**) Transwell assay assessing the effect of RhELNs on HAEC migration under hypoxia. (**D**) Quantitative analysis of Transwell assay results. (**E**) Scratch assay evaluating HAEC migration capacity following RhELNs treatment under hypoxia. (**F**) Quantitative analysis of the scratch assay. (**G, H**) TUNEL staining for detecting HAEC apoptosis and quantitative analysis. *P < 0.05, **P < 0.01, ***P < 0.001.

The 5-Ethynyl-2′-deoxyuridine (EdU) assay demonstrated a dose-dependent increase in cells under hypoxic conditions following RhELNs treatment (Figure 2A and B). Transwell and scratch assays both showing enhanced migratory capacity under hypoxia (Figure 2C–F). Additionally, TUNEL assay showed that RhELNs alleviated apoptosis in HAEC under hypoxic conditions, and their anti-apoptotic ability was enhanced with increasing concentrations of RhELNs (Figure 2G and H). Overall, these results suggest that RhELNs promote proliferation, migration, and invasion of vascular endothelial cells while mitigating hypoxia-induced apoptosis *in vitro*.

### RhELNs could reduce the inflammatory level and promote the tissue repair of ischemic skeletal muscle

**Figure 3.**
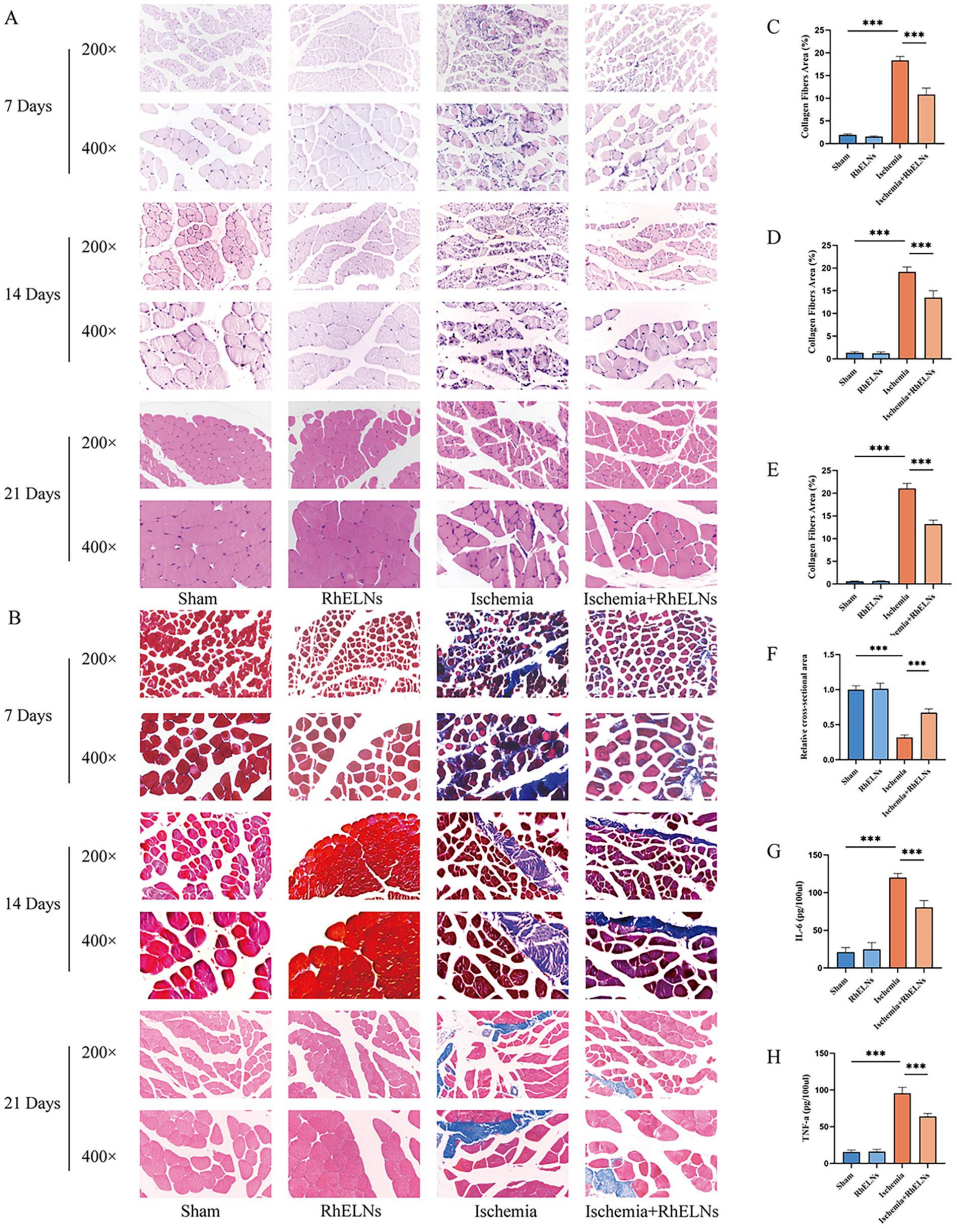
Histological and cytokine alterations in skeletal muscle post-RhELNs treatment in a lower limb skeletal muscle ischemia model. (**A**) HE staining of skeletal muscle at 7 days, 14 days and 21 days after surgery. (**B**) Masson trichrome staining of skeletal muscles at 7 days, 14 days and 21 days after surgery. (**C**) Quantitative histological analysis of Masson staining at 7 days after surgery. (**D**) Quantitative histological analysis of Masson staining at 14 days after surgery. (**E**) Quantitative histological analysis of Masson staining at 21 days after surgery. (**F**) Comparison of skeletal muscle cross-sectional area relative to the Sham group at 21 days post-surgery. (**G, H**) Levels of TNF-α and IL-6 in skeletal muscle 7 days post-surgery. *n* ≥ 3 for each group at each time point. *P < 0.05, **P < 0.01, ***P < 0.001.

To evaluate the potential of RhELNs in repairing lower limb ischemia, a hind limb ischemia model was established. The experimental groups included the Sham group, RhELNs group (tail vein injection of RhELNs following sham surgery), Ischemia group (femoral artery ligation), and Ischemia+RhELNs group (femoral artery ligation followed by RhELNs injection). HE staining of muscle tissue revealed a marked reduction in inflammatory cell infiltration within skeletal muscle following RhELNs treatment compared to the ischemia group (Figure 3A). Similarly, Masson’s trichrome staining showed that after RhELN administration, fibrosis in the skeletal muscles decreased remarkably at various time points (Figure 3B–E). In addition, by quantitatively comparing the cross-sectional area of the skeletal muscles in mice stained with HE, it can be observed that after 21 days of RhELNs treatment, the muscle atrophy caused by limb ischemia in mice has been obvious alleviated (Figure 3F). Additionally, ELISA results of tissue samples demonstrated that RhELNs effectively reduced levels of pro-inflammatory cytokines IL-6 and TNF-α in post-ischemic muscle tissue (Figure 3G and H). ELISA results of mouse serum samples illustrated that RhELNs did not cause abnormal levels of CRP, IL-1β and IL-8 in mouse serum (Supplementary Figure S7). Furthermore, histological examination of the heart, liver, spleen, lung and kidney on day 14 showed no abnormalities in the RhELNs-treated mice compared to the Sham group, suggesting that RhELNs exhibited good *in vivo* biocompatibility (Supplementary Figure S10).

### RhELNs can promote blood perfusion and angiogenesis in the lower limb skeletal muscle ischemia model

**Figure 4.**
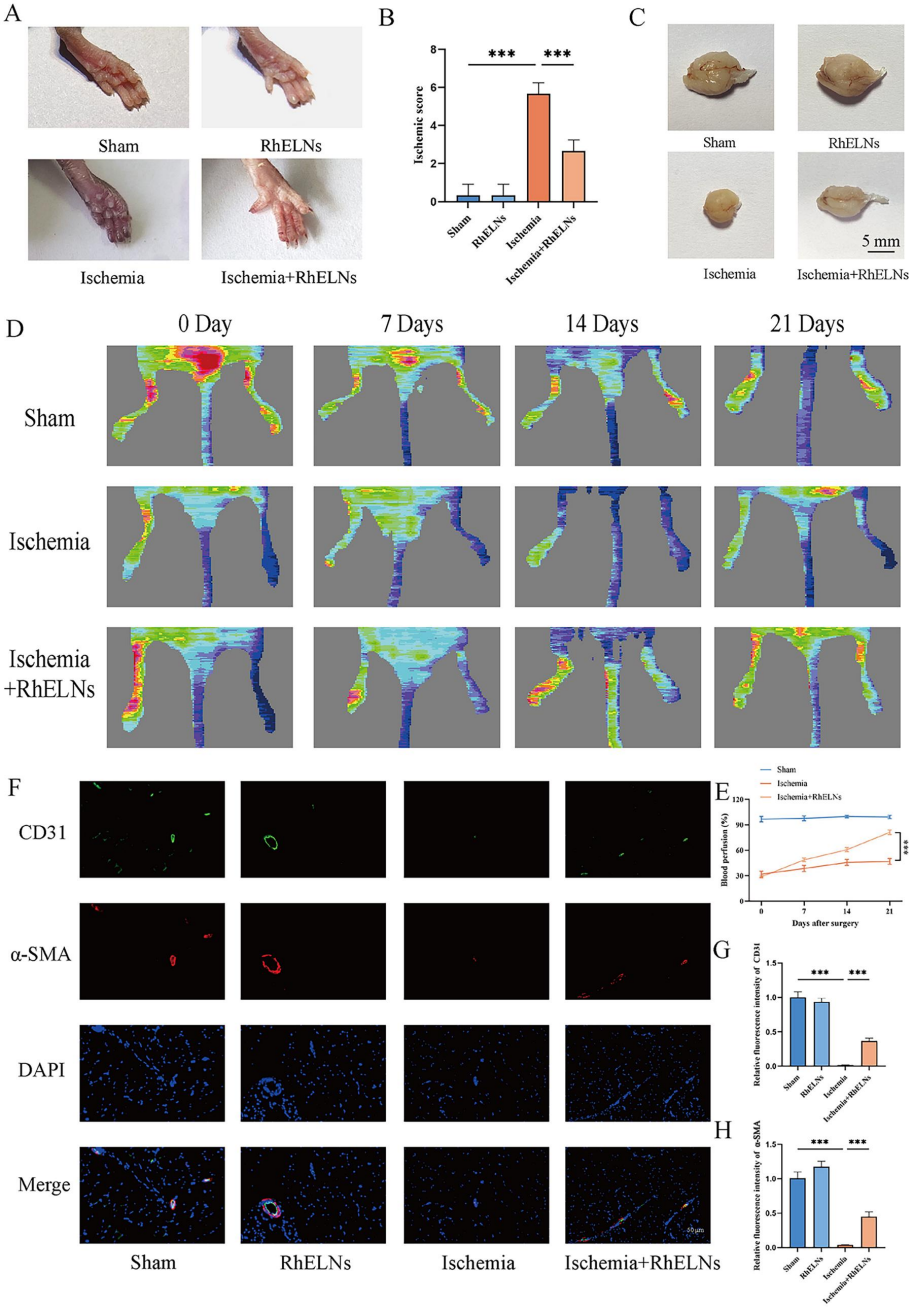
Effects of RhELNs on perfusion and angiogenesis in a lower limb skeletal muscle ischemia model. (**A**) Gross appearance of the lower limbs at 14 days post-surgery. (**B**) Limb ischemia score. (**C**) Gross appearance of skeletal muscle at 14 days post-surgery. (**D**) Limb perfusion measurements at 0, 7, 14 and 21 days post-surgery. (**E**) Quantitative analysis of limb perfusion ratios across the different groups. (**F**) Immunofluorescence staining of CD31 and α-SMA in skeletal muscle 14 days post-surgery. (**G, H**) Quantitative analysis of immunofluorescence staining for CD31 and α-SMA. *n*≥3 for each group at each time point. *P < 0.05, **P < 0.01, ***P < 0.001.

Fourteen days post-surgery, photographs of the lower limbs revealed significant stasis and gangrene in the Ischemia group compared to the Sham group, while RhELNs-treated mice showed marked improvement in ischemic symptoms (Figure 4A). To assess the severity of limb ischemia in mice, we referred to the ischemia scoring method used by Wong et al. [22]. Quantification of scores reveals that RhELNs contribute to the treatment of ischemic injury in the lower limb of mice (Figure 4B). By day 14, skeletal muscle atrophy observed in the Ischemia group was notably attenuated in RhELNs-treated mice, indicating improved muscle preservation (Figure 4C). Doppler flowmetry indicated that the perfusion ratios in RhELNs-treated mice were higher than those in untreated mice at 7, 14 and 21 days, relative to the contralateral limb with normal blood flow (Figure 4D and E). Immunofluorescence analysis of muscle tissues at day 14 showed significantly increased expression of CD31 and α-SMA in RhELNs-treated ischemic limbs compared to the untreated ischemic group, indicating enhanced neovascularization capacity following RhELNs treatment (Figure 4F–H). These results indicate that RhELNs not only reduce inflammation and fibrosis at the ischemic site but also facilitate blood vessel formation and maturation. Overall, the results suggest that RhELNs play a critical role in promoting the repair and recovery of lower limb ischemia by enhancing vascular regeneration and mitigating ischemia-related tissue damage.

### Novel-miRNA-115-5p in RhELNs can target and regulate TXNIP expression

**Figure 5.**
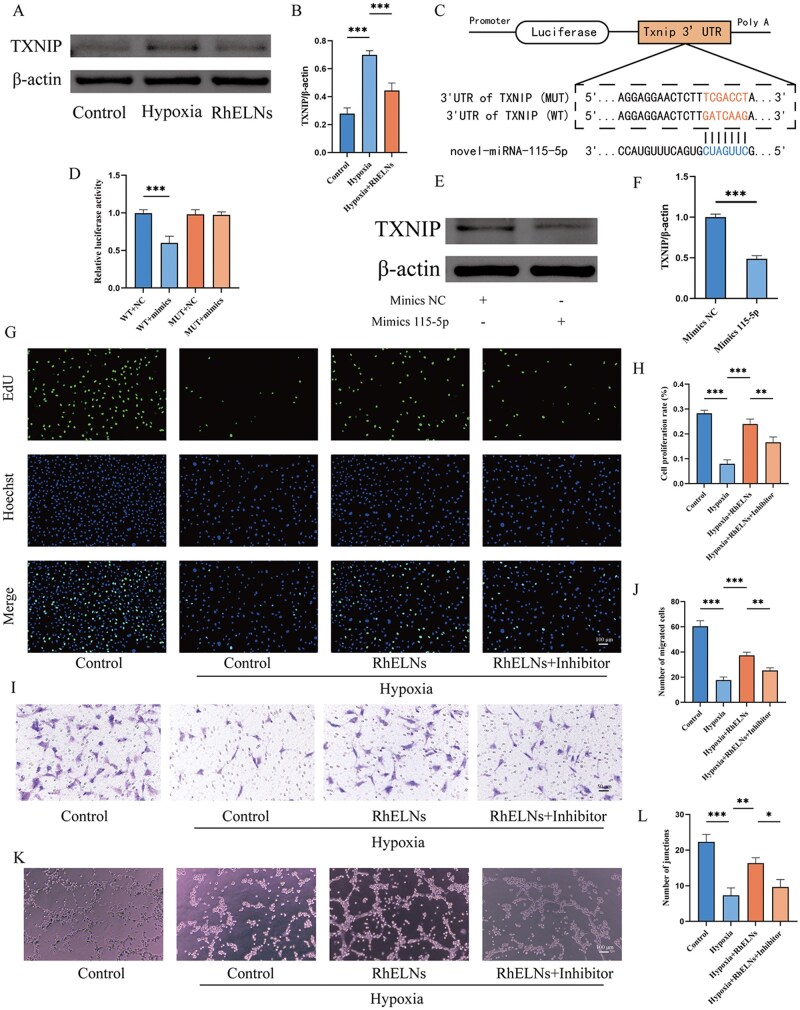
Regulation of TXNIP expression by novel-miRNA-115-5p in RhELNs. (**A, B**) Western blot experiments demonstrated that RhELNs inhibited TXNIP expression under hypoxia and corresponding quantitative data. (**C**) Analysis of the binding site between novel-miRNA-115-5p and TXNIP, including the mutated sequence, using miRanda software. (**D**) Dual luciferase reporter assay confirming the interaction between novel-miRNA-115-5p and TXNIP. (**E, F**) Western blot analysis of TXNIP protein levels in HAEC and corresponding quantitative data. (**G**) EdU assay results for HAEC proliferation across different treatment groups. (**H**) Quantitative analysis of the EdU assay. (**I**) Transwell assay results assessing HAEC migration in each group. (**J**) Quantitative analysis of the Transwell assay. (**K**) Tubule formation assay for angiogenesis evaluation in different groups. (**L**) Quantitative analysis of the tubule formation assay. *P < 0.05, **P < 0.01, ***P < 0.001.

Western blot demonstrated the inhibitory effect of RhELNs on TXNIP expression in HAECs under hypoxia (Figure 5A and B). Further, a novel miRNA was identified in RhELNs: novel-miRNA-115-5p (miRNA sequence: GCUUGAUCGUGACUUUGUACC). MiRanda software analysis predicted that novel-miRNA-115-5p could target the 3′UTR of TXNIP, suggesting its potential role in regulating biological functions by inhibiting TXNIP expression. To confirm TXNIP as a direct target of novel-miRNA-115-5p, a dual luciferase reporter assay was performed, demonstrating a targeting interaction between novel-miRNA-115-5p and TXNIP (Figure 5C and D). Further validation showed that transfection of novel-miRNA-115-5p mimics into HAECs led to a significant decrease in TXNIP protein levels (Figure 5E and F). To explore the functional role of novel-miRNA-115-5p in RhELNs, assays for cell proliferation (EdU assay, Figure 5G and H), migration (Transwell assay, Figure 5I and J) and angiogenesis (tubule formation assay, Figure 5K and L) were conducted. Under hypoxic conditions, HAECs exhibited reduced proliferative, migratory, and angiogenic capacities. However, treatment with RhELNs significantly restored these functions, while inhibition of novel-miRNA-115-5p reversed the protective effects of RhELNs. These results suggest that RhELNs confer vascular endothelial protection by delivering novel-miRNA-115-5p, which modulates key cellular processes through the suppression of TXNIP expression.

### novel-miRNA-115-5p regulates HAEC proliferation and migration

**Figure 6.**
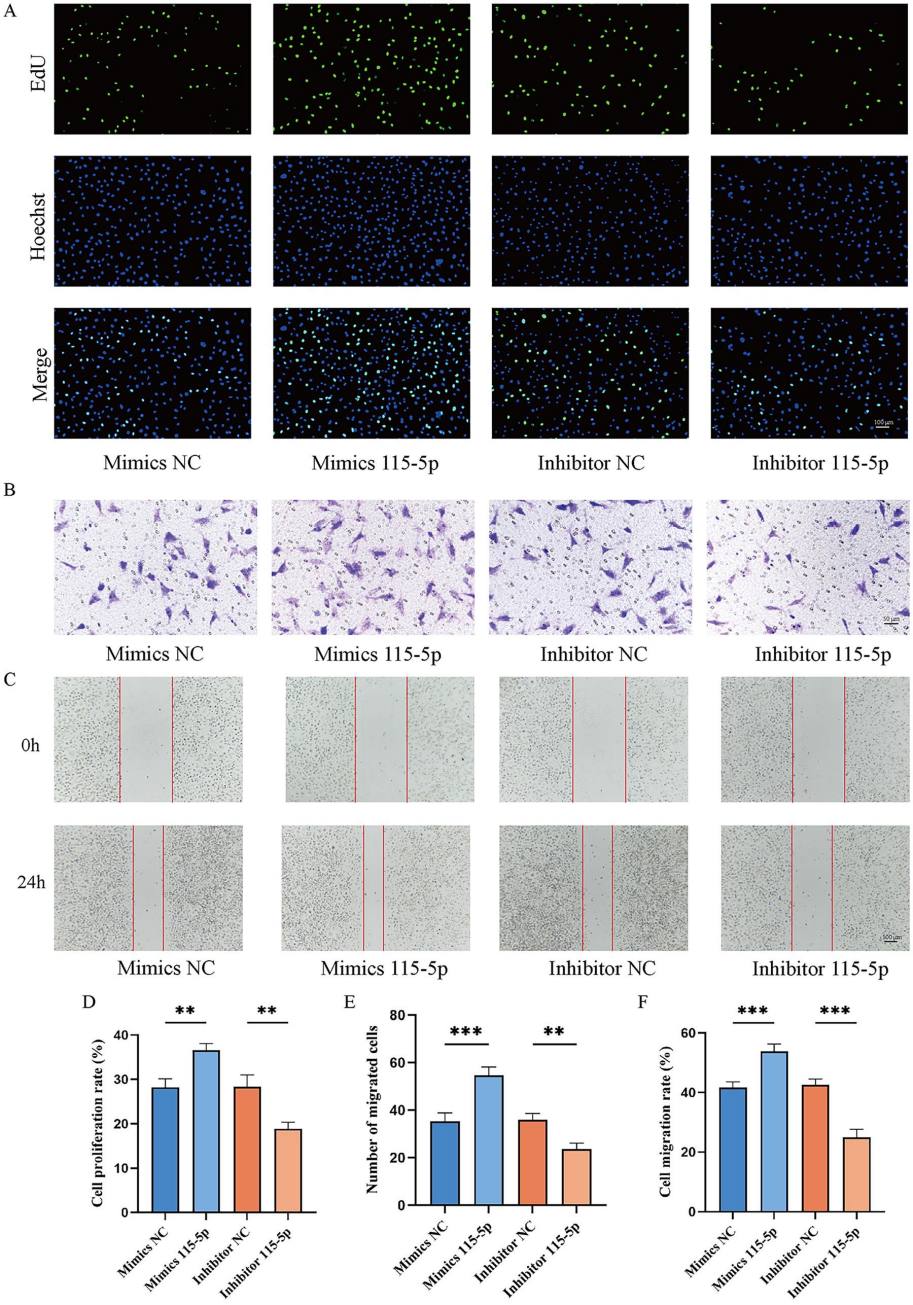
Regulation of HAEC proliferation and migration by novel-miRNA-115-5p. (**A**) EdU assay results showing the proliferation rates of HAEC across four groups. (**B**) Transwell assay results illustrating HAEC migration rates in the four groups. (**C**) Scratch assay results showing migration rates of HAEC in the four groups. (**D**) Quantitative analysis of EdU assay data. (**E**) Quantitative analysis of Transwell assay results. (**F**) Quantitative analysis of scratch assay results. *P < 0.05, **P < 0.01, ***P < 0.001.

To investigate the biological effects of novel-miRNA-115-5p, HAECs under hypoxic conditions were transfected with novel-miRNA-115-5p mimics or inhibitors. The EdU assay demonstrated that cell proliferation was significantly enhanced by the mimics, whereas the inhibitor reduced proliferation, indicating that novel-miRNA-115-5p plays a role in regulating cell growth (Figure 6A and D). Similarly, the Transwell and scratch wound healing assays showed that novel-miRNA-115-5p promoted HAEC migration, while the inhibitor had the opposite effect (Figure 6B, C, E, and F). These results confirm that novel-miRNA-115-5p mimics enhance cell proliferation and migration, whereas the inhibitor exerts inhibitory effects.

### novel-miRNA-115-5p/TXNIP axis regulates vascular endothelial cell function

**Figure 7.**
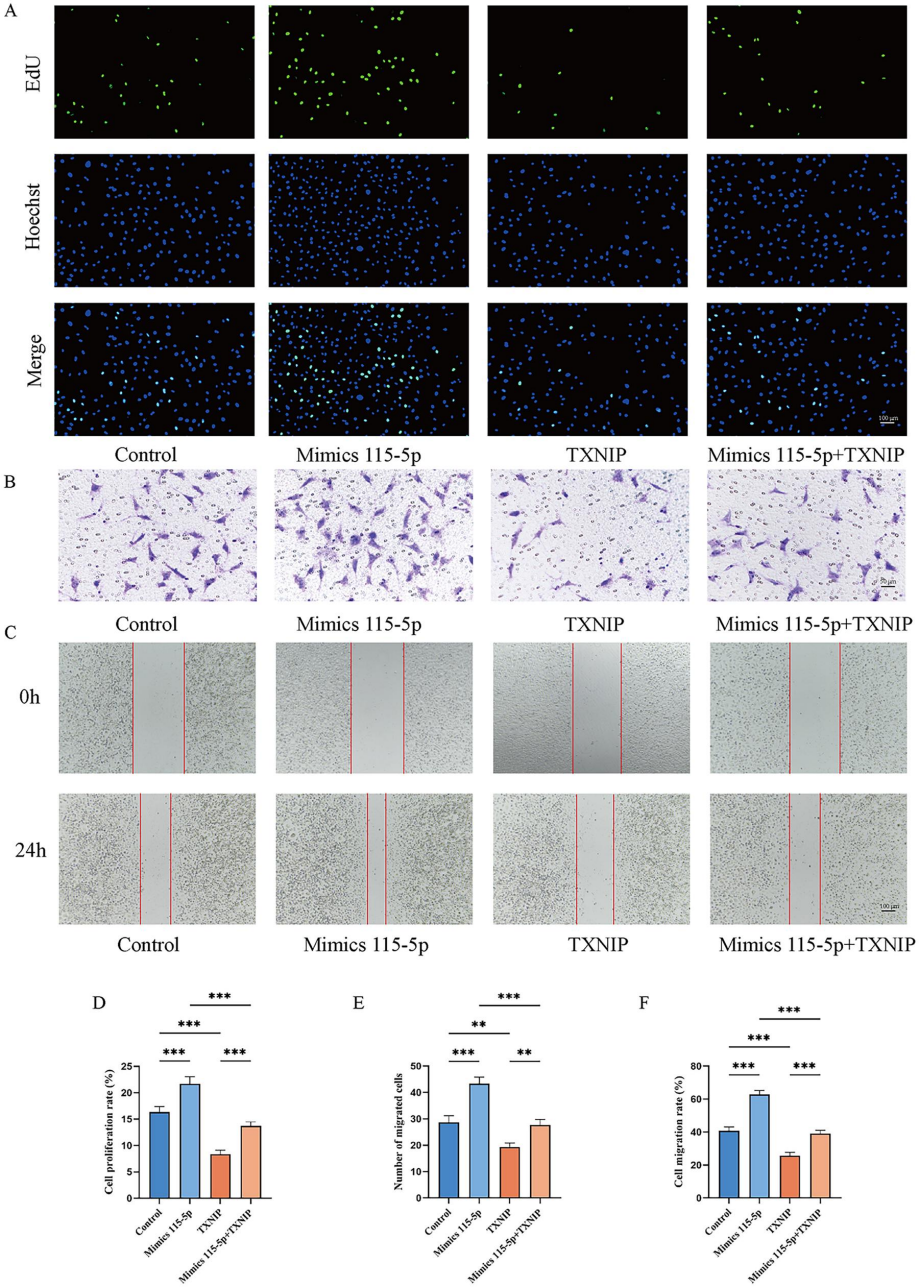
Regulation of vascular endothelial cell function by the novel-miRNA-115-5p/TXNIP axis. (**A**) EdU assay evaluating HAEC proliferation in different treatment groups. (**B**) Transwell assay assessing HAEC migration capacity in different treatment groups. (**C**) Scratch assay evaluating the migration ability of HAEC across various treatment groups. (**D**) Quantitative analysis of EdU assay results. (**E**) Quantitative analysis of Transwell assay data. (**F**) Quantitative analysis of scratch assay results. *P < 0.05, **P < 0.01, ***P < 0.001.

To further explore the function of the novel-miRNA-115-5p/TXNIP axis, TXNIP was overexpressed in hypoxic HAECs *via* transfection with a TXNIP expression plasmid. The HAECs under hypoxic conditions were used as the control group. The EdU assay showed that TXNIP overexpression significantly suppressed cell proliferation, counteracting the proliferative effects of novel-miRNA-115-5p mimics (Figure 7A and D). In the Transwell assay, TXNIP overexpression also inhibited cell migration, negating the promotive effect of novel-miRNA-115-5p mimics (Figure 7B and E). The scratch assay results were consistent with these findings (Figure 7C and F). These data suggest that novel-miRNA-115-5p modulates the proliferative and migratory capacities of vascular endothelial cells through the TXNIP axis.

### The novel-miRNA-115-5p/TXNIP axis regulates vascular endothelial cell death

**Figure 8.**
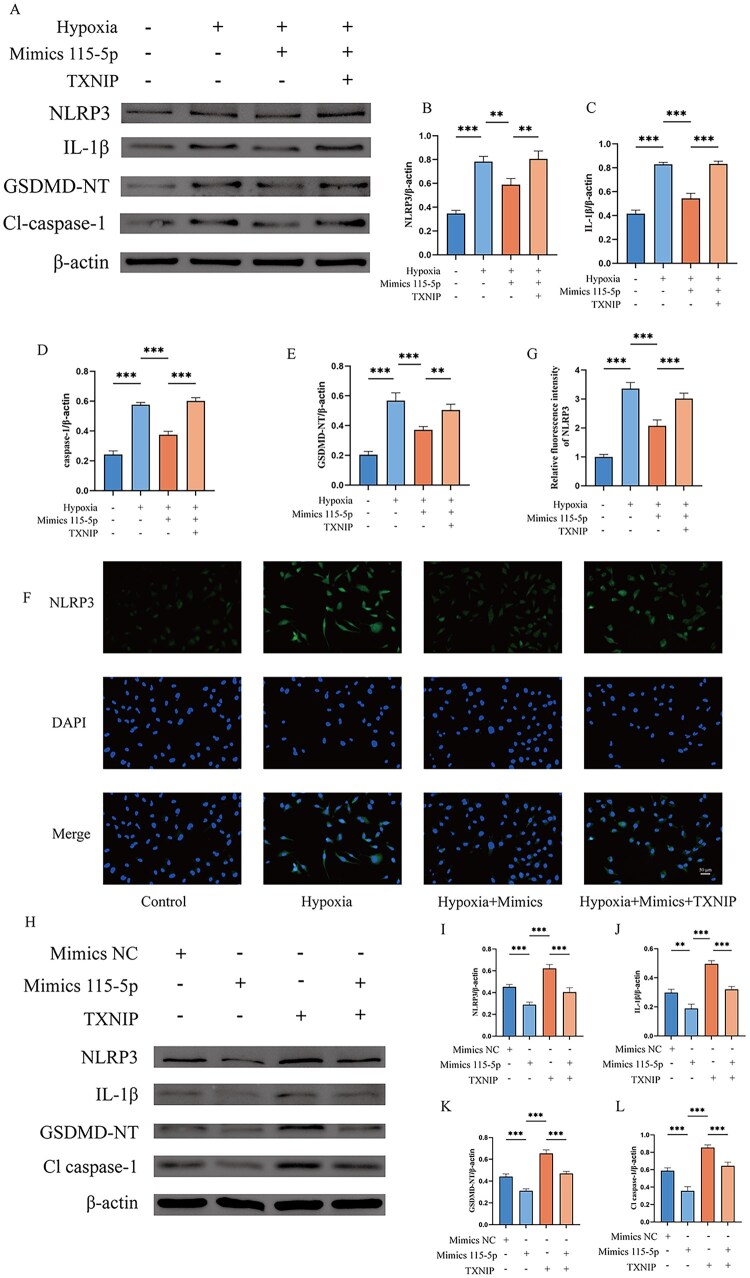
Regulation of vascular endothelial cell pyroptosis by the novel-miRNA-115-5p/TXNIP axis. (**A–E**) Western blot experiments were conducted to detect the effects of different treatment groups on the expression levels of proteins related to pyroptosis of cells and the quantitative analysis of protein expression levels. (**F, G**) Immunofluorescence and quantitative analysis of fluorescence expression of NLRP3. (**H–L**) Western blot experiments and quantitative analysis of protein expression levels of the rescue experiment demonstrating the inhibitory effect of novel miRNA-115-5p on pyroptotic damage in hypoxia-injured vascular endothelial cells. *P < 0.05, **P < 0.01, ***P < 0.001.

Hypoxia-induced cell death in vascular endothelial cells often involve the NLRP3 inflammasome, which serves as a key mediator in cell death pathways. Activation of the NLRP3 inflammasome leads to cleavage of GSDMD, producing GSDMD-NT, which forms membrane pores and promotes the release of inflammatory mediators. TXNIP is a key regulator of NLRP3 activation. The results indicated that transfection with novel-miRNA-115-5p mimics reduced the elevated levels of key proteins associated with hypoxia-induced pyroptosis, including NLRP3, GSDMD-NT, cleaved caspase-1 and IL-1β (Figure 8A–E). Simultaneously, we further validated the role of the novel miRNA-115-5p/TXNIP axis in regulating vascular endothelial cell death by comparing the inhibitory effects of novel miRNA-115-5p and Mimics NC on pyroptosis and by overexpressing TXNIP (Figure 8F–L). These results highlight the role of the novel-miRNA-115-5p/TXNIP axis in modulating the cellular response to hypoxic stress, including proliferation, migration, and pyroptosis in vascular endothelial cells. Furthermore, we used novel-miRNA-115-5p to treat lower limb ischemia mice, and demonstrated that novel-miRNA-115-5p could repair lower limb skeletal muscle ischemic injury by relieving the NLRP3-mediated pyroptosis pathway through western blot experiments at the animal level (Supplementary Figure S9).

### The novel-miRNA-115-5p/TXNIP axis protects against mitochondrial damage in vascular endothelial cells

**Figure 9.**
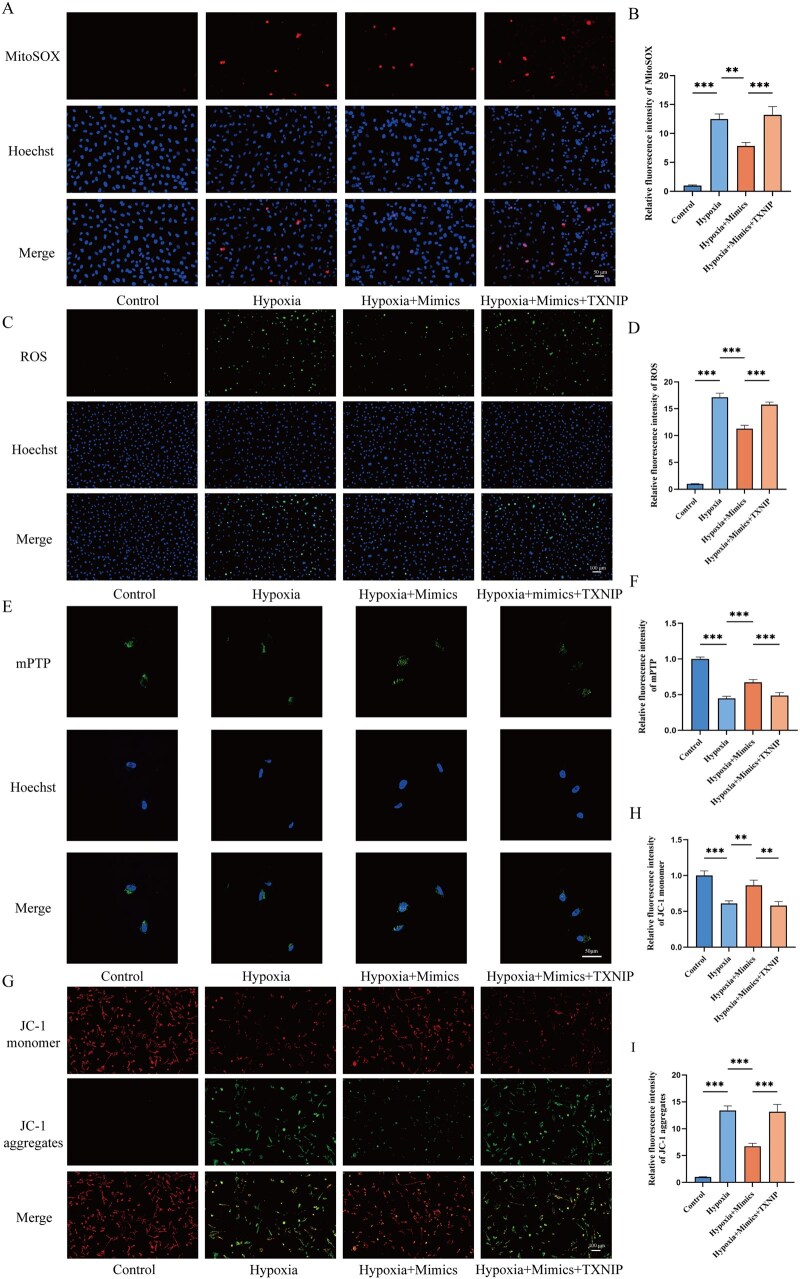
Impact of the novel-miRNA-115-5p/TXNIP axis on mitochondrial damage in vascular endothelial cells. (**A, B**) MitoSOX fluorescence staining images of cells from different treatment groups and quantitative analysis. (**C, D**) ROS levels in cells under various treatment conditions and quantitative analysis. (**E, F**) Fluorescence images representing mPTP openness in cells from different treatment groups and quantitative analysis. (**G**) Mitochondrial membrane potential measurements in cells across different treatment groups and quantitative analysis. (**H**) Quantitative analysis of fluorescence intensity of JC-1monomer in different treatment groups. (**I**) Quantitative analysis of fluorescence intensity of JC-1 aggregates from different treatment groups. *P < 0.05, **P < 0.01, ***P < 0.001.

Given the significant role of TXNIP in regulating mitochondrial biological functions, we evaluated mtROS levels, ROS levels, mitochondrial permeability transition pore (mPTP) open levels, and Mitochondrial membrane potential (MMP) in these cells. The results showed that hypoxia significantly increased mtROS levels, ROS levels, mPTP open levels in vascular endothelial cells and disrupted MMP. However, treatment with the novel miRNA-115-5p mimics reduced mtROS production (Figure 9A and B), inhibited cellular ROS accumulation (Figure 9C and D), reduced mPTP open levels (Figure 9E and F) and improved MMP stability (Figure 9G–I). When TXNIP was overexpressed, the beneficial effect of novel-miRNA-115-5p mimic was reversed, confirming that novel-miRNA-115-5p protected cells from mitochondrial damage under hypoxia by targeting TXNIP.

## Discussion

In recent years, extracellular vesicles have gained significant attention as a novel therapeutic modality complementary to traditional medicine. Released by a variety of cell types, including both animal and plant cells, these vesicles play a pivotal role in intra- and intercellular communication by transporting bioactive molecules [23–25].

Since the initial discovery of mammalian extracellular vesicles (MEVs), extensive research has characterized their secretion, uptake mechanisms, physical properties, composition, and roles in various pathophysiological processes [24, 26, 27]. However, the clinical application of MEVs is limited by the complex culture requirements of mammalian cells, stringent purification standards, high production costs, and low yields [7, 28]. Consequently, attention has shifted toward plant-derived exosome-like nanovesicles, which have shown promise in numerous studies for their anti-infective, anti-inflammatory, anti-tumor, anti-aging and regenerative properties [29–34].

Rhodiola rosea, a natural ‘medicine and food’ plant, contains an array of bioactive substances including vitamins, phenolic compounds, proteins, amino acids, sterols, organic acids, lipids, glycosides and trace elements [35, 36]. Known for its resilience and adaptability in harsh environments, Rhodiola rosea has demonstrated potential *in vitro*, non-clinical, and clinical studies for combating fatigue, enhancing exercise endurance, and treating conditions such as hypertension, ischemic diseases and COVID-19 [11, 37–39].

In this study, exosome-like nanovesicles were successfully extracted from fresh Rhodiola rosea rhizomes using sucrose gradient centrifugation. Characterization confirmed that these RhELNs exhibited typical exosome features and demonstrated good stability. Co-culture experiments with vascular endothelial cells showed efficient cellular uptake of RhELNs without significant cytotoxicity.

Considering that vascular injury repair and regeneration is crucial in lower limb ischemia, we referred to previous studies to treat lower limb ischemia by tail vein injection [40, 41]. RhELNs could act on the injured blood vessels through the systemic circulation and promote the regeneration of blood vessels to treat lower limb ischemic injury.

In a lower limb skeletal muscle ischemia model, RhELNs treatment improved muscle perfusion, promoted neovascularization, and alleviated skeletal muscle atrophy and gangrene. Histopathological analysis of skeletal muscle tissue revealed a significant reduction in inflammatory cell infiltration and decreased fibrosis following RhELN administration, indicating their therapeutic potential for ischemic conditions.

MiRNAs from plant-derived ELNs play a significant role in regulating gene expression and modulating receptor functions, thus influencing various physiological and pathological processes in animals. For example, miRNAs in ginger-derived ELNs help maintain gut microbiota balance, while those in honey-derived ELNs mitigate inflammation by targeting NLRP3 [42, 43]. Additionally, Rgl-exomiR-7972 from groundnut ELNs has been shown to alleviate Lipopolysaccharide (LPS)-induced acute lung injury [44].

This study further explored the function of novel-miRNA-115-5p in RhELNs, which targets TXNIP. Inhibition of novel-miRNA-115-5p was found to reduce the capacity of Rhodiola rosea-derived ELNs to enhance vascular endothelial cell proliferation, migration, and angiogenesis, highlighting its critical role in the biological activity of RhELNs. Dual luciferase reporter and western blot assays confirmed that novel-miRNA-115-5p suppresses TXNIP expression, while overexpression of TXNIP reversed the beneficial effects of novel-miRNA-115-5p on HAEC proliferation, migration, and angiogenesis.

TXNIP is a widely expressed protein activated by cellular stresses such as inflammation and oxidative stress, facilitating the release of pro-inflammatory cytokines [15]. It is integral to the regulation of angiogenesis, inflammatory responses, and metabolic processes, and modulates vascular endothelial cell proliferation and migration by influencing intracellular oxidative stress. Lowering TXNIP expression in endothelial cells is essential for protecting against arterial injury [18, 45].

Cellular pyroptosis, a form of cysteine-dependent pro-inflammatory cell death characterized by cell swelling and membrane lysis, can be triggered through the activation of TXNIP-NLRP3 signaling [46, 47]. This process is associated with the release of inflammatory mediators such as IL-1β, which amplifies local inflammation and promotes apoptosis [15, 45, 48].

In this study, novel-miRNA-115-5p was shown to inhibit the expression of NLRP3, GSDMD-NT, and IL-1β under hypoxic conditions, indicating its ability to reduce vascular endothelial cell pyroptosis and suppress the release of inflammatory factors. As the main oxygen-consuming organelles, mitochondria are the first to be affected under ischemic conditions. During ischemia and hypoxia, damaged mitochondria release a large amount of superoxide, resulting in a large amount of ROS. At the same time, excessive ROS will stimulate the excessive opening of mPTP, which will lead to the change of mitochondrial membrane potential, further cause mitochondrial damage, and eventually lead to cell death. TXNIP plays an important role in regulating the production of cellular mitochondrial reactive oxygen species (mtROS). So in this study, we explored the protective effect of novel-miRNA-115-5p on mitochondrial damage under hypoxia by regulating the expression of TXNIP.

The findings suggest that Rhodiola rosea-derived exosome-like nanovesicles offer protective effects against lower limb ischemic injury. Specifically, they can alleviate vascular endothelial cell pyroptosis under hypoxia by inhibiting the TXNIP-NLRP3 signaling pathway *via* novel-miRNA-115-5p, and which can also protect against mitochondrial injury in vascular endothelial cells by inhibiting the expression of TXNIP, contributing to the treatment of skeletal muscle ischemia in the lower limbs. This study offers new insights into the therapeutic potential of Rhodiola rosea-derived exosome-like nanovesicles and elucidates their underlying mechanisms of action. Considering that vascular endothelial cells are susceptible to ischemia and hypoxia, the damage and repair of vascular endothelial cells play a crucial role in the progression of limb ischemic diseases. Therefore, in this study, we primarily focused on the damage repair effect of RhELNs on vascular endothelial cells. Meanwhile, macrophages or other cells also play significant roles in the progression of limb ischemic diseases. Our current research still has limitations. In future research, we will further explore whether RhELNs can regulate the biological functions of macrophages and other cells.

## Conclusion

Exosome-like nanovesicles derived from fresh Rhodiola rosea rhizomes were successfully extracted, and their therapeutic potential was demonstrated. *In vivo* studies showed that Rhodiola rosea-derived ELNs significantly inhibited the progression of lower limb ischemia, mitigated skeletal muscle atrophy, and suppressed the release of inflammatory factors. *In vitro* experiments further revealed that novel-miRNA-115-5p, identified within RhELNs, protected vascular endothelial cells from mitochondrial by targeting TXNIP, and promote vascular regeneration by reducing cell pyroptosis under hypoxic conditions through inhibition of the TXNIP-NLRP3 pathway. These results suggest that RhELN administration represents a promising new approach for treating lower limb skeletal muscle ischemic diseases.

## Supplementary Material

rbaf113_Supplementary_Data
