# Supplementary material for: Rhodiola rosea-derived exosome-like nanovesicles inhibit vascular endothelial pyroptosis in the treatment of limb skeletal muscle ischemic injury through the TXNIP/NLNP3 pathway
Source: Regen Biomater. 2025 Oct 31;12:rbaf113. doi: 10.1093/rb/rbaf113 (PMC12681252; doi:10.1093/rb/rbaf113)
Supplement: rbaf113_Supplementary_Data [file rbaf113_supplementary_data.zip › Supplementary Table S1.docx]

**Table S1** Primary metabolites of RhELNs.

| **No.** | **Name** | **Class** | **Formula** | **Molecularweight(Da)** |
| --- | --- | --- | --- | --- |
| 1 | MG(18:2/0:0/0:0) | Lipids​ | C21H38O4 | 354.277 |
| 2 | 1-Stearoyl-sn-glycerol | Lipids​ | C21H42O4 | 358.3083 |
| 3 | 1,2-Dioctanoyl-sn-glycerol | Lipids​ | C19H36O5 | 344.2563 |
| 4 | [3-hydroxy-2-[3,4,5-trihydroxy-6-(hydroxymethyl)oxan-2-yl]oxypropyl] (7Z,10Z,13Z)-hexadeca-7,10,13-trienoate | Lipids​ | C25H42O9 | 486.2829 |
| 5 | 1-(9Z-tetradecenoyl)-2-hexadecanoyl-sn-glycerol | Lipids​ | C33H62O5 | 538.4597 |
| 6 | 1,2-Dihexanoyl-sn-glycerol | Lipids​ | C15H28O5 | 288.1937 |
| 7 | [(2S)-2-[(9Z,12Z)-octadeca-9,12-dienoyl]oxy-3-[(2R,3R,4S,5R,6R)-3,4,5-trihydroxy-6-[[(2R,3R,4S,5R,6R)-3,4,5-trihydroxy-6-(hydroxymethyl)oxan-2-yl]oxymethyl]oxan-2-yl]oxypropyl] (9Z,12Z)-octadeca-9,12-dienoate | Lipids​ | C51H88O15 | 940.6123 |
| 8 | TG(14:0/16:1(9Z)/22:1(13Z)) | Lipids​ | C55H102O6 | 858.7676 |
| 9 | [(2S)-1-hexadecanoyloxy-3-[(2R,3R,4S,5R,6R)-3,4,5-trihydroxy-6-[[(2R,3R,4S,5R,6R)-3,4,5-trihydroxy-6-(hydroxymethyl)oxan-2-yl]oxymethyl]oxan-2-yl]oxypropan-2-yl] (9Z,12Z,15Z)-octadeca-9,12,15-trienoate | Lipids​ | C49H86O15 | 914.5967 |
| 10 | TG(15:0/20:0/15:0) | Lipids​ | C53H102O6 | 834.7676 |
| 11 | [(2S)-1-[(9E,12Z,15Z)-octadeca-9,12,15-trienoyl]oxy-3-[(2R,3R,4S,5R,6R)-3,4,5-trihydroxy-6-[[(2R,3R,4S,5R,6R)-3,4,5-trihydroxy-6-(hydroxymethyl)oxan-2-yl]oxymethyl]oxan-2-yl]oxypropan-2-yl] (9Z,12Z)-octadeca-9,12-dienoate | Lipids​ | C51H86O15 | 938.5967 |
| 12 | [(2S)-2-[(9Z,12E,15Z)-octadeca-9,12,15-trienoyl]oxy-3-[(2R,3R,4S,5R,6R)-3,4,5-trihydroxy-6-[[(2R,3R,4S,5R,6R)-3,4,5-trihydroxy-6-(hydroxymethyl)oxan-2-yl]oxymethyl]oxan-2-yl]oxypropyl] (9Z,12Z,15Z)-octadeca-9,12,15-trienoate | Lipids​ | C51H84O15 | 936.581 |
| 13 | PA(18:3(9Z,12Z,15Z)/20:1(11Z)) | Lipids​ | C41H73O8P | 724.5043 |
| 14 | (2R)-2-hydroxy-3-(phosphonooxy)propyl hexadecanoate | Lipids​ | C19H39O7P | 410.2433 |
| 15 | 1-(11Z-octadecenoyl)-2-(15Z-tetracosenoyl)-sn-glycero-3-phosphocholine | Lipids​ | C50H96NO8P | 869.6874 |
| 16 | 1,2-Dimyristoyl-sn-glycero-3-phosphocholine | Lipids​ | C36H72NO8P | 677.4996 |
| 17 | 1-Vaccenoyl-2-pentadecanoyl-sn-glycero-3-phosphocholine | Lipids​ | C41H80NO8P | 745.5622 |
| 18 | 1-docosanoyl-2-(9Z-tetradecenoyl)-glycero-3-phosphoethanolamine | Lipids​ | C41H80NO8P | 745.5622 |
| 19 | 1-Hexadecanoyl-2-pentadecanoyl-glycero-3-phosphoethanolamine | Lipids​ | C36H72NO8P | 677.4996 |
| 20 | LysoPA(18:2(9Z,12Z)/0:0) | Lipids​ | C21H39O7P | 434.2433 |
| 21 | Glycerophosphoethanolamine | Lipids​ | C5H14NO6P | 215.0559 |
| 22 | LysoPE(0:0/16:0) | Lipids​ | C21H44NO7P | 453.2855 |
| 23 | FFA(18:2) | Lipids​ | C18H32O2 | 280.2402 |
| 24 | FFA(12:0) | Lipids​ | C12H24O2 | 200.1776 |
| 25 | FAA(18:1) | Lipids​ | C18H34O2 | 282.2559 |
| 26 | FFA(16:1) | Lipids​ | C16H30O2 | 254.2246 |
| 27 | Docosatrienoic acid | Lipids​ | C22H38O2 | 334.2872 |
| 28 | (S)-10,16-Dihydroxyhexadecanoic acid | Lipids​ | C16H32O4 | 288.2301 |
| 29 | Eicosadienoic acid | Lipids​ | C20H36O2 | 308.2715 |
| 30 | FFA(20:0) | Lipids​ | C20H40O2 | 312.3028 |
| 31 | 16-Hydroxyhexadecanoic acid | Lipids​ | C16H32O3 | 272.2351 |
| 32 | gamma-Linolenic Acid | Lipids​ | C18H30O2 | 278.2246 |
| 33 | (R)-10-hydroxystearic acid | Lipids​ | C18H36O3 | 300.2664 |
| 34 | Lignoceric acid | Lipids​ | C24H48O2 | 368.3654 |
| 35 | Tricosanoic acid | Lipids​ | C23H46O2 | 354.3498 |
| 36 | FFA(19:1) | Lipids​ | C19H36O2 | 296.2715 |
| 37 | alpha-Linolenic acid | Lipids​ | C18H30O2 | 278.2246 |
| 38 | FFA(20:1) | Lipids​ | C20H38O2 | 310.2872 |
| 39 | L-Valine | Amino acids and derivatives | C5H11NO2 | 117.079 |
| 40 | L-Isoleucine | Amino acids and derivatives | C6H13NO2 | 131.0946 |
| 41 | Asparaginyl-Leucine | Amino acids and derivatives | C10H19N3O4 | 245.1376 |
| 42 | Phosphoserine | Amino acids and derivatives | C3H8NO6P | 185.0089 |
| 43 | His-Ala-Gln | Amino acids and derivatives | C14H22N6O5 | 354.1652 |
| 44 | Val-Leu | Amino acids and derivatives | C11H22N2O3 | 230.163 |
| 45 | L-Proline | Amino acids and derivatives | C5H9NO2 | 115.0633 |
| 46 | Asn-Lys-Phe-Asp | Amino acids and derivatives | C23H34N6O8 | 522.2438 |
| 47 | Methionine | Amino acids and derivatives | C5H11NO2S | 149.051 |
| 48 | L-Leucyl-L-alanine | Amino acids and derivatives | C9H18N2O3 | 202.1317 |
| 49 | Val-Glu | Amino acids and derivatives | C10H18N2O5 | 246.1216 |
| 50 | Gln-Leu | Amino acids and derivatives | C11H21N3O4 | 259.1532 |
| 51 | Histidylleucine | Amino acids and derivatives | C12H20N4O3 | 268.1535 |
| 52 | Glu-Leu | Amino acids and derivatives | C11H20N2O5 | 260.1372 |
| 53 | Glycyl-L-leucine | Amino acids and derivatives | C8H16N2O3 | 188.1161 |
| 54 | Leu-Ile | Amino acids and derivatives | C12H24N2O3 | 244.1787 |
| 55 | L-Tyrosine | Amino acids and derivatives | C9H11NO3 | 181.0739 |
| 56 | Val-Ile-Glu | Amino acids and derivatives | C16H29N3O6 | 359.2056 |
| 57 | Glu-Ile-Asp | Amino acids and derivatives | C15H25N3O8 | 375.1642 |
| 58 | H-Leu-val-OH | Amino acids and derivatives | C11H22N2O3 | 230.163 |
| 59 | Phe-Glu | Amino acids and derivatives | C14H18N2O5 | 294.1216 |
| 60 | Ile-Glu-Asn | Amino acids and derivatives | C15H26N4O7 | 374.1801 |
| 61 | Ile-Gly-Glu | Amino acids and derivatives | C13H23N3O6 | 317.1587 |
| 62 | Arg-Leu | Amino acids and derivatives | C12H25N5O3 | 287.1957 |
| 63 | Ile-Gln | Amino acids and derivatives | C11H21N3O4 | 259.1532 |
| 64 | 2-[(2-Amino-3-hydroxybutanoyl)amino]-3-phenylpropanoic acid | Amino acids and derivatives | C13H18N2O4 | 266.1267 |
| 65 | His-Gln-Ala | Amino acids and derivatives | C14H22N6O5 | 354.1652 |
| 66 | Pro-Phe-Ile | Amino acids and derivatives | C20H29N3O4 | 375.2158 |
| 67 | Leu-Glu | Amino acids and derivatives | C11H20N2O5 | 260.1372 |
| 68 | Valylserine | Amino acids and derivatives | C8H16N2O4 | 204.111 |
| 69 | Thr-Leu | Amino acids and derivatives | C10H20N2O4 | 232.1423 |
| 70 | L-Phenylalanine | Amino acids and derivatives | C9H11NO2 | 165.079 |
| 71 | Leu-Asn | Amino acids and derivatives | C10H19N3O4 | 245.1376 |
| 72 | Thr-Met | Amino acids and derivatives | C9H18N2O4S | 250.0987 |
| 73 | Asn-Phe | Amino acids and derivatives | C13H17N3O4 | 279.1219 |
| 74 | Thr-Lys-Asn | Amino acids and derivatives | C14H27N5O6 | 361.1961 |
| 75 | Adenosine | Nucleotides and derivatives | C10H13N5O4 | 267.0968 |
| 76 | Adenine | Nucleotides and derivatives | C5H5N5 | 135.0545 |
| 77 | Guanosine | Nucleotides and derivatives | C10H13N5O5 | 283.0917 |
| 78 | Uridine | Nucleotides and derivatives | C9H12N2O6 | 244.0695 |
| 79 | Guanine | Nucleotides and derivatives | C5H5N5O | 151.0494 |
| 80 | Adenosine monophosphate | Nucleotides and derivatives | C10H14N5O7P | 347.0631 |
| 81 | Cytosine | Nucleotides and derivatives | C4H5N3O | 111.0433 |
| 82 | Cytidine | Nucleotides and derivatives | C9H13N3O5 | 243.0855 |
| 83 | Uridine-5'-monophosphate | Nucleotides and derivatives | C9H13N2O9P | 324.0359 |
| 84 | 5,6-dihydrothymine | Nucleotides and derivatives | C5H8N2O2 | 128.0586 |
| 85 | 5,6-dihydrothymine | Nucleotides and derivatives | C5H8N2O2 | 128.0586 |
| 86 | 2'-Deoxyadenosine-5'-monophosphate | Nucleotides and derivatives | C10H14N5O6P | 331.0682 |
| 87 | 5'-phosphoribosyl-N-formylglycinamide | Nucleotides and derivatives | C8H15N2O9P | 314.0515 |
| 88 | Xanthosine | Nucleotides and derivatives | C10H12N4O6 | 284.0757 |
| 89 | 2'-Deoxyadenosine | Nucleotides and derivatives | C10H13N5O3 | 251.1018 |
| 90 | Uracil | Nucleotides and derivatives | C4H4N2O2 | 112.0273 |
| 91 | Inosine | Nucleotides and derivatives | C10H12N4O5 | 268.0808 |
| 92 | [(2R,5R)-5-(4-amino-2-oxopyrimidin-1-yl)-4-hydroxy-2-(hydroxymethyl)oxolan-3-yl] dihydrogen phosphate | Nucleotides and derivatives | C9H14N3O8P | 323.0519 |
| 93 | Sedoheptulose | Saccharides and their derivatives | C7H14O7 | 210.074 |
| 94 | alpha-D-Glucose | Saccharides and their derivatives | C6H12O6 | 180.0634 |
| 95 | L-glyceraldehyde | Saccharides and their derivatives | C3H6O3 | 90.0317 |
| 96 | D-(-)-Fructose | Saccharides and their derivatives | C6H12O6 | 180.0634 |
| 97 | Cellobiose | Saccharides and their derivatives | C12H22O11 | 342.1162 |
| 98 | Sucrose | Saccharides and their derivatives | C12H22O11 | 342.1162 |
| 99 | Alpha-Maltose | Saccharides and their derivatives | C12H22O11 | 342.1162 |
| 100 | 2-Deoxy-D-ribose | Saccharides and their derivatives | C5H10O4 | 134.0579 |
| 101 | Glucosamine | Saccharides and their derivatives | C6H13NO5 | 179.0794 |
| 102 | Gluconic acid | Saccharides and their derivatives | C6H12O7 | 196.0583 |
| 103 | Glucose | Saccharides and their derivatives | C6H12O6 | 180.0634 |
| 104 | D-galactaro-1,4-lactone | Saccharides and their derivatives | C6H8O7 | 192.027 |
| 105 | Succinic acid | Organic acids | C4H6O4 | 118.0266 |
| 106 | D-Malic acid | Organic acids | C4H6O5 | 134.0215 |
| 107 | cis-Aconitic acid | Organic acids | C6H6O6 | 174.0164 |
| 108 | L-Lactic acid | Organic acids | C3H6O3 | 90.0317 |
| 109 | Citric acid | Organic acids | C6H8O7 | 192.027 |
| 110 | Oxoglutaric acid | Organic acids | C5H6O5 | 146.0215 |
| 111 | Pyruvic acid | Organic acids | C3H4O3 | 88.016 |
| 112 | Glutaric acid | Organic acids | C5H8O4 | 132.0423 |
| 113 | 2-Phospho-D-Glyceric Acid | Organic acids | C3H7O7P | 185.9929 |
| 114 | 2-Hydroxyhexadecanoic acid | Organic acids | C16H32O3 | 272.2351 |
| 115 | Malic acid | Organic acids | C4H6O5 | 134.0215 |
| 116 | Phosphoenolpyruvate | Organic acids | C3H5O6P | 167.9824 |
| 117 | Glyceric acid | Organic acids | C3H6O4 | 106.0266 |
| 118 | Pyridoxine | Others | C8H11NO3 | 169.0739 |
| 119 | Niacinamide | Others | C6H6N2O | 122.048 |
