# Supplementary material for: Rhodiola rosea-derived exosome-like nanovesicles inhibit vascular endothelial pyroptosis in the treatment of limb skeletal muscle ischemic injury through the TXNIP/NLNP3 pathway
Source: Regen Biomater. 2025 Oct 31;12:rbaf113. doi: 10.1093/rb/rbaf113 (PMC12681252; doi:10.1093/rb/rbaf113)
Supplement: rbaf113_Supplementary_Data [file rbaf113_supplementary_data.zip › Supplementary Table S2.docx]

**Table S2** Secondary metabolites of RhELNs.

| **No.** | **Name** | **Class** | **Formula** | **Molecularweight(Da)** |
| --- | --- | --- | --- | --- |
| 1 | Salidroside | Phenols and Derivatives​ | C14H20O7 | 300.1209 |
| 2 | 6-O-Galloylglucose | Phenols and Derivatives​ | C13H16O10 | 332.0743 |
| 3 | Caffeic acid | Phenols and Derivatives​ | C9H8O4 | 180.0423 |
| 4 | Gallic acid | Phenols and Derivatives​ | C7H6O5 | 170.0215 |
| 5 | 1-Phenylethanol | Phenols and Derivatives​ | C8H10O | 122.0732 |
| 6 | 1,2,3-Trihydroxybenzene | Phenols and Derivatives​ | C6H6O3 | 126.0317 |
| 7 | Pyrocatechol | Phenols and Derivatives​ | C6H6O2 | 110.0368 |
| 8 | Phenol | Phenols and Derivatives​ | C6H6O | 94.0419 |
| 9 | 3,4-Dimethylbenzoic acid | Phenols and Derivatives​ | C9H10O2 | 150.0681 |
| 10 | Iriflophenone 3-C-glucoside | Phenols and Derivatives​ | C19H20O10 | 408.1056 |
| 11 | 2-Hydroxycinnamic acid | Phenols and Derivatives​ | C9H8O3 | 164.0473 |
| 12 | 4-(Hydroxymethyl)-2-methylphenol | Phenols and Derivatives​ | C8H10O2 | 138.0681 |
| 13 | Cinnamic acid | Phenols and Derivatives​ | C9H8O2 | 148.0524 |
| 14 | Chlorogenic Acid | Phenols and Derivatives​ | C16H18O9 | 354.0951 |
| 15 | Ethyl gallate | Phenols and Derivatives​ | C9H10O5 | 198.0528 |
| 16 | Caffeyl alcohol | Phenols and Derivatives​ | C9H10O3 | 166.063 |
| 17 | p-Coumaryl alcohol | Phenols and Derivatives​ | C9H10O2 | 150.0681 |
| 18 | Rhodiolin | Phenols and Derivatives​ | C25H20O10 | 480.1056 |
| 19 | Octyl gallate | Phenols and Derivatives​ | C15H22O5 | 282.1467 |
| 20 | 3,4-Dimethoxybenzoic acid | Phenols and Derivatives​ | C9H10O4 | 182.0579 |
| 21 | p-Coumaraldehyde | Phenols and Derivatives​ | C9H8O2 | 148.0524 |
| 22 | Ferulic acid | Phenols and Derivatives​ | C10H10O4 | 194.0579 |
| 23 | 1,6-bis-O-galloyl-beta-D-glucose | Phenols and Derivatives​ | C20H20O14 | 484.0853 |
| 24 | Thymol | Phenols and Derivatives​ | C10H14O | 150.1045 |
| 25 | 1,2,3,6-Tetrakis-O-galloyl-beta-D-glucose | Phenols and Derivatives​ | C34H28O22 | 788.1072 |
| 26 | 1,2,6-Trigalloylglucose | Phenols and Derivatives​ | C27H24O18 | 636.0963 |
| 27 | 4-Hydroxycinnamic acid | Phenols and Derivatives​ | C9H8O3 | 164.0473 |
| 28 | Salicylic acid | Phenols and Derivatives​ | C7H6O3 | 138.0317 |
| 29 | 4-Methoxybenzaldehyde | Phenols and Derivatives​ | C8H8O2 | 136.0524 |
| 30 | (−)-Rosavin | Phenols and Derivatives​ | C20H28O10 | 428.16795 |
| 31 | 4-Hydroxy-3-methoxybenzenemethanol | Phenols and Derivatives​ | C8H10O3 | 154.063 |
| 32 | 1-O-trans-Cinnamoyl-beta-D-glucopyranose | Phenols and Derivatives​ | C15H18O7 | 310.1053 |
| 33 | 3-Hydroxycinnamic acid | Phenols and Derivatives​ | C9H8O3 | 164.0473 |
| 34 | Guaiacol | Phenols and Derivatives​ | C7H8O2 | 124.0524 |
| 35 | 3,5-Dihydroxyanisole | Phenols and Derivatives​ | C7H8O3 | 140.0473 |
| 36 | Tyrosol | Phenols and Derivatives​ | C8H10O2 | 138.0681 |
| 37 | 1-O-feruloyl-beta-D-glucose | Phenols and Derivatives​ | C16H20O9 | 356.1107 |
| 38 | 4-[(1S)-1-hydroxyethyl]phenol | Phenols and Derivatives​ | C8H10O2 | 138.0681 |
| 39 | Sinapic acid | Phenols and Derivatives​ | C11H12O5 | 224.0685 |
| 40 | Sakuranetin | Flavonoids | C16H14O5 | 286.0841 |
| 41 | Naringenin | Flavonoids | C15H12O5 | 272.0685 |
| 42 | Taxifolin | Flavonoids | C15H12O7 | 304.0583 |
| 43 | Luteolin | Flavonoids | C15H10O6 | 286.0477 |
| 44 | Gallocatechin-(4alpha->8)-epigallocatechin | Flavonoids | C30H26O14 | 610.1323 |
| 45 | (-)-Gallocatechin | Flavonoids | C15H14O7 | 306.074 |
| 46 | Kaempferol | Flavonoids | C15H10O6 | 286.0477 |
| 47 | Quercetin | Flavonoids | C15H10O7 | 302.0427 |
| 48 | Rutin | Flavonoids | C27H30O16 | 610.1534 |
| 49 | Morin | Flavonoids | C15H10O7 | 302.0427 |
| 50 | Quercitrin | Flavonoids | C21H20O11 | 448.1006 |
| 51 | Rhamnetin | Flavonoids | C16H12O7 | 316.0583 |
| 52 | (+)-Gallocatechin | Flavonoids | C15H14O7 | 306.074 |
| 53 | Epigallocatechin | Flavonoids | C15H14O7 | 306.074 |
| 54 | Epigallocatechin gallate | Flavonoids | C22H18O11 | 458.0849 |
| 55 | Epigallocatechin-(4beta->8)-epicatechin-3-O-gallate ester | Flavonoids | C37H30O17 | 746.1483 |
| 56 | (3E,6Z)-3,6-Nonadien-1-ol | Alcohols | C9H16O | 140.1201 |
| 57 | (-)-Dihydrocarveol | Alcohols | C10H18O | 154.1358 |
| 58 | (1S,2R,4R)-Neoiso-dihydrocarveol | Alcohols | C10H18O | 154.1358 |
| 59 | (-)-Neoisodihydrocarveol | Alcohols | C10H18O | 154.1358 |
| 60 | (1S)-6-Hydroxy-1,7,7-trimethylbicyclo[2.2.1]heptan-2-one | Alcohols | C10H16O2 | 168.115 |
| 61 | (1R,2S,4R)-Neo-dihydrocarveol | Alcohols | C10H18O | 154.1358 |
| 62 | (-)-trans-Isopiperitenol | Alcohols | C10H16O | 152.1201 |
| 63 | 3,7-Dimethyl-1,6-octadien-3-ol | Alcohols | C10H18O | 154.1358 |
| 64 | Trigonelline | Alkaloids | C7H7NO2 | 137.0477 |
| 65 | Dopamine | Alkaloids | C8H11NO2 | 153.079 |
| 66 | Theobromine | Alkaloids | C7H8N4O2 | 180.0647 |
| 67 | Tyramine | Alkaloids | C8H11NO | 137.0841 |
| 68 | Indole | Alkaloids | C8H7N | 117.0578 |
| 69 | Serotonin | Alkaloids | C10H12N2O | 176.095 |
| 70 | Nootkatone | Terpenoids | C15H22O | 218.1671 |
| 71 | Rishitin | Terpenoids | C14H22O2 | 222.162 |
| 72 | (-)-Oleoside 11-methyl ester | Terpenoids | C17H24O11 | 404.1319 |
| 73 | Geraniol | Terpenoids | C10H18O | 154.1358 |
| 74 | trans-Verbenol | Terpenoids | C10H16O | 152.1201 |
| 75 | Ellagic acid | Others | C14H6O8 | 302.0063 |
| 76 | Theaflavin monogallates | Others | C36H28O16 | 716.1377 |
| 77 | Matairesinol | Others | C20H22O6 | 358.1416 |
| 78 | Demethylwedelolactone | Others | C15H8O7 | 300.027 |
| 79 | Embelin | Others | C17H26O4 | 294.1831 |
