# Supplementary material for: Rhodiola rosea-derived exosome-like nanovesicles inhibit vascular endothelial pyroptosis in the treatment of limb skeletal muscle ischemic injury through the TXNIP/NLNP3 pathway
Source: Regen Biomater. 2025 Oct 31;12:rbaf113. doi: 10.1093/rb/rbaf113 (PMC12681252; doi:10.1093/rb/rbaf113)
Supplement: rbaf113_Supplementary_Data [file rbaf113_supplementary_data.zip › Supplementary Document.docx]

gcatgtggaagaaaagaagcagctttacctacttgtttctttttgtctctcttcctggacactcactttttcagagactcaacagtctctgcaatggagtgtgggtccaccttagcctctgacttcctaatgtaggaggtggtcagcaggcaatctcctgggccttaaaggatgcggactcatcctcagccagcgcccatgttgtgatacaggggtgtttgttggatgggtttaaaaataactagaaaaactcagggccatccattttctcagatctccttgaaaattgaggccttttcgatagtttcgggtcaggtaaaatggcctcctggcgtaagcttttcaaggttttttggaggctttttgtaaattgtgataggaactttggaccttgaacttacgtatcatgtggagaagagccaanttaacaaactaggaagatgaaaagggaaattgtggccaaaactttgggaaaaggaggttcttaaaatcagtgtttcccctttgtgcacttgtagaaaaaaaagaaaaaccttctagagctgatttgatggacaatggagagagctttccctgtgattataaaaaaggaagctagctgctctacggtcatctttgcttagagtatactttaacctggcttttaaagcagtagtaactgccccaccaaaggtcttaaaagccatttttggagcctattgcactgtgttctcctactgcaaatattttcatatgggaggatggttttctcttcatgtaagtccttggaattgattctaaggtgatgttcttagcactttaattcctgtcaaattttttgttctcccttctgccatcttaaatgtaagctgaaactggtctactgtgtctctagggttaagcaaaagacaaaaaaaattttactacttttgagattgccccaatgtacagaattatataattctaacgcttaaatcatgtgaaagggttgctgctgtcagccttgcccactgtgacttcaaacccaaggaggaactcttgatcaagatgcccaaccctgtgatcagaacctccaaatactgccatgagaaactagagggcaggtcttcataaaagccctttgaacccccttcctgccctgtgttaggagatagggatattggcccctcactgcagctgccagcacttggtcagtcactctcagccatagcactttgttcactgtcctgtgtcagagcactgagctccacccttttctgagagttattacagccagaaagtgtgggctgaagatggttggtttcatgtttttgtattatgtatctttttgtatggtaaagactatattttgtacttaaccagatatatttttaccccagatggggatattctttgtaaaaaatgaaaataaagtttttttaatggaaaaaaaaaa

**Data S1.** 3'UTR sequence of TXNIP (yellow marked portion is the binding site of novel-miRNA-115-5p)


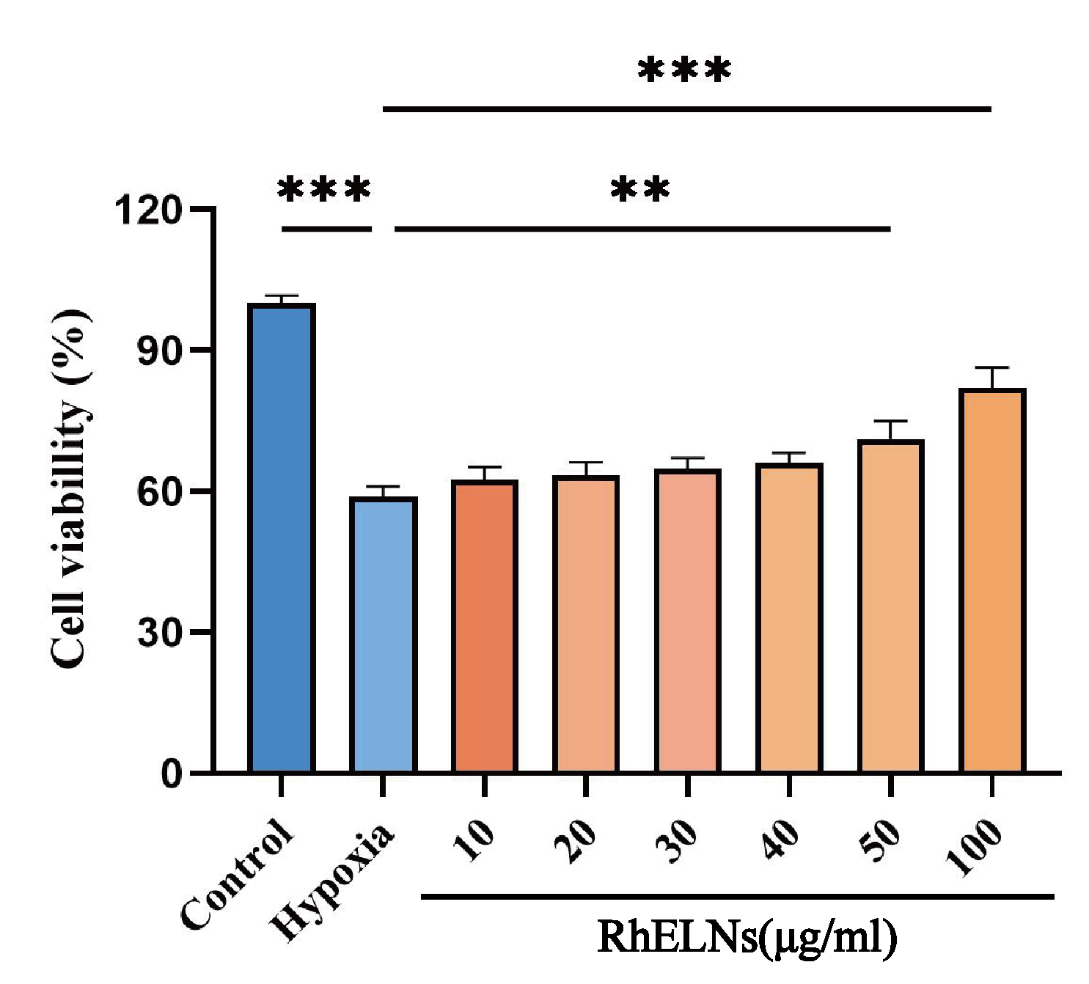


**Figure S1.** CCK-8 of RhELNs.


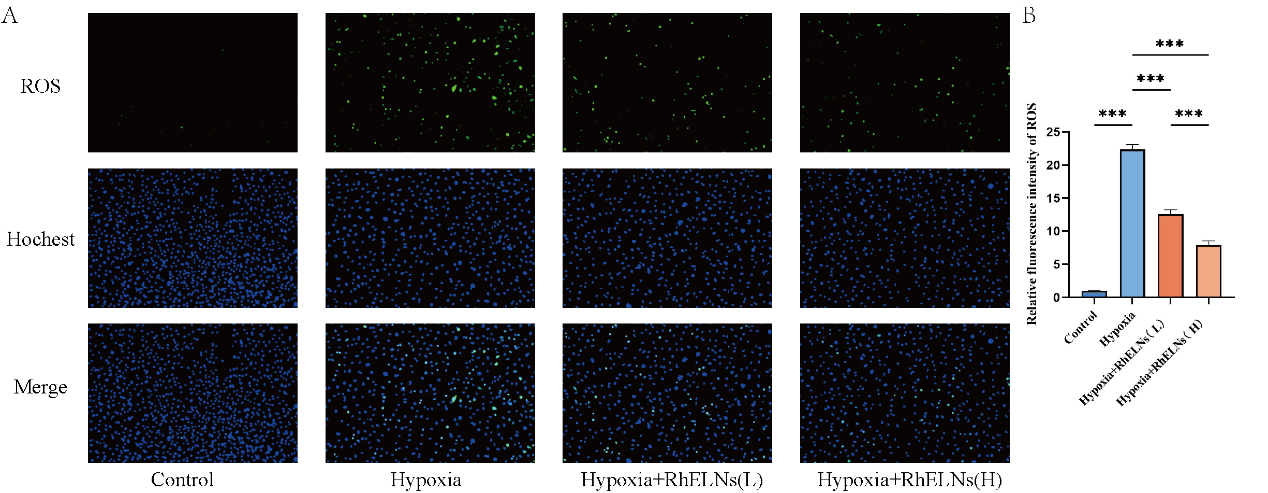


**Figure S2.** Regulation of ROS levels in vascular endothelial cells by RhELNs.

(A) ROS fluorescence expression levels in cells after different treatments. (B) Quantitative analysis of ROS fluorescence expression levels in cells after different treatments.


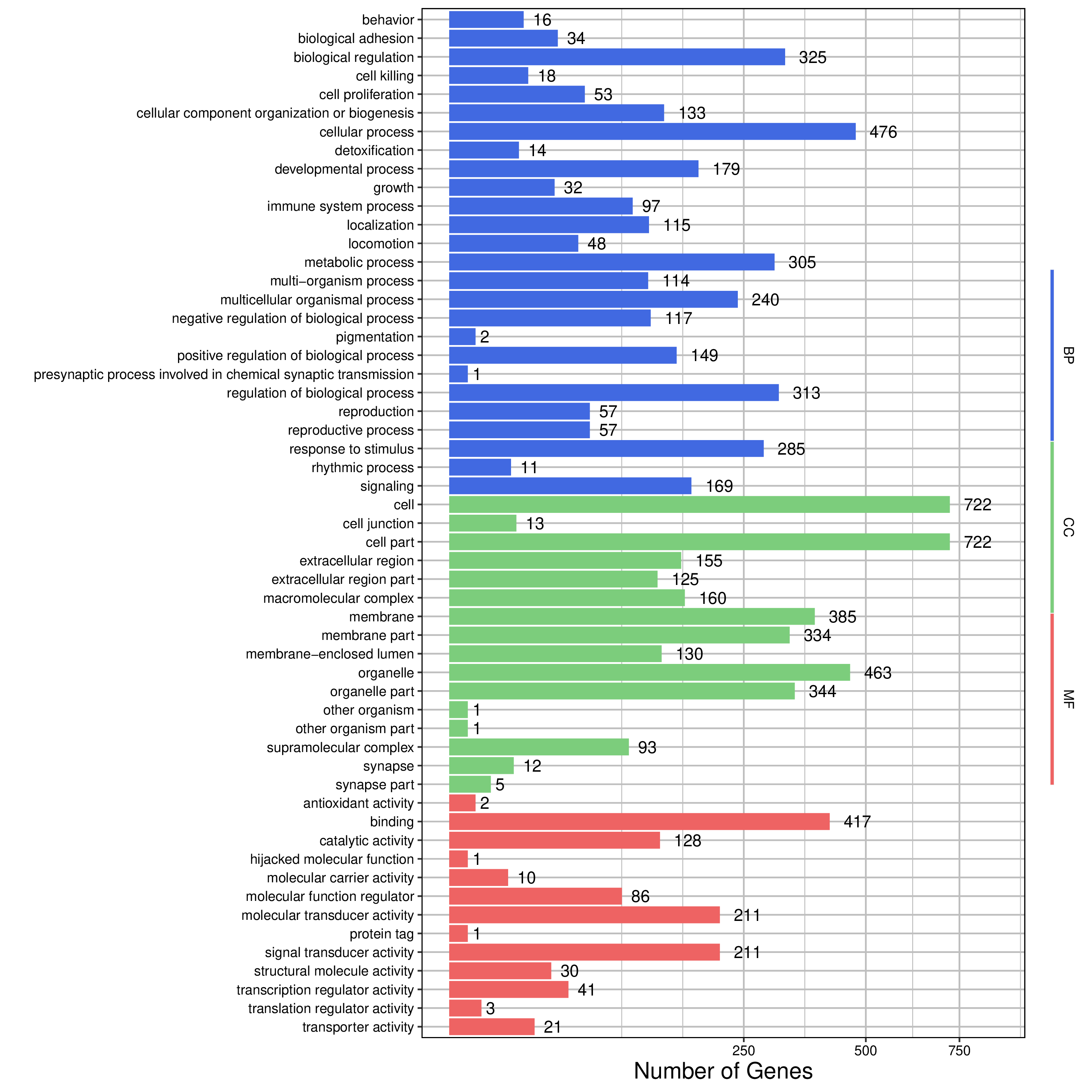


**Figure S3.** GO enrichment analysis of miRNA target genes of RhELNs.


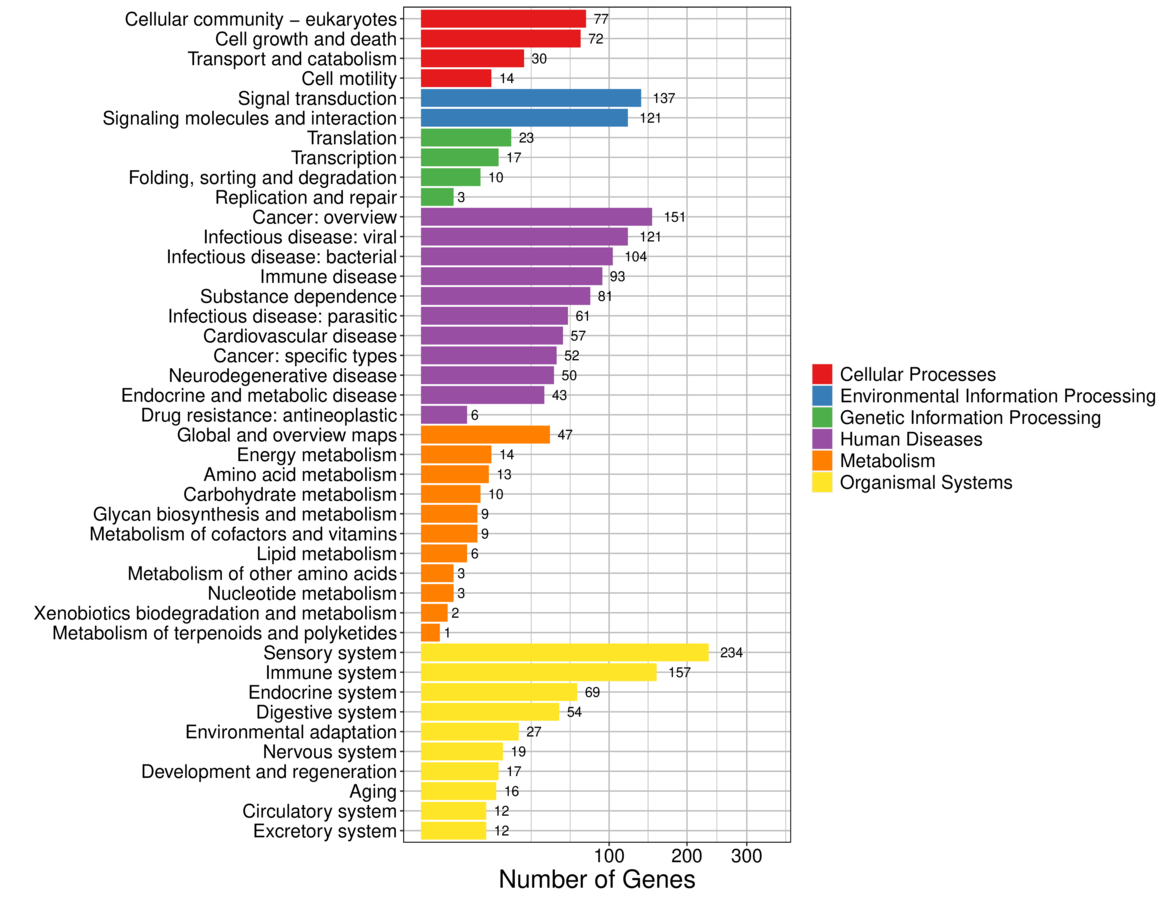


**Figure S4.** Analysis of KEGG enrichment of miRNA target genes for RhELNs.


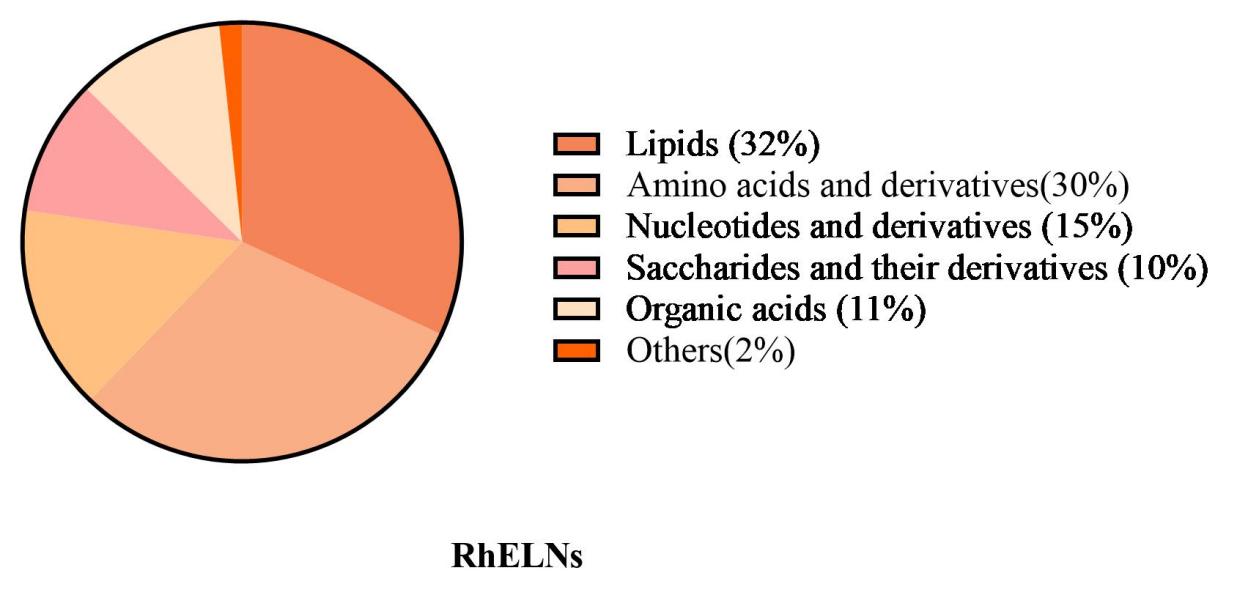


**FigureS5**. Primary metabolite substance analysis in RhELNs.


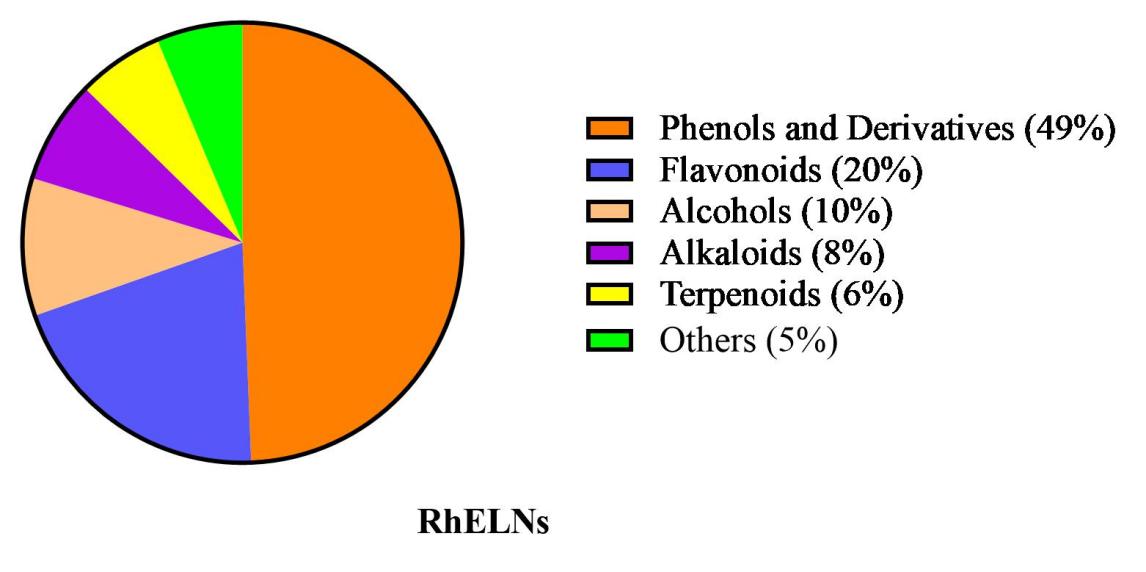


**FigureS6.** Material analysis of secondary metabolites in RhELNs.


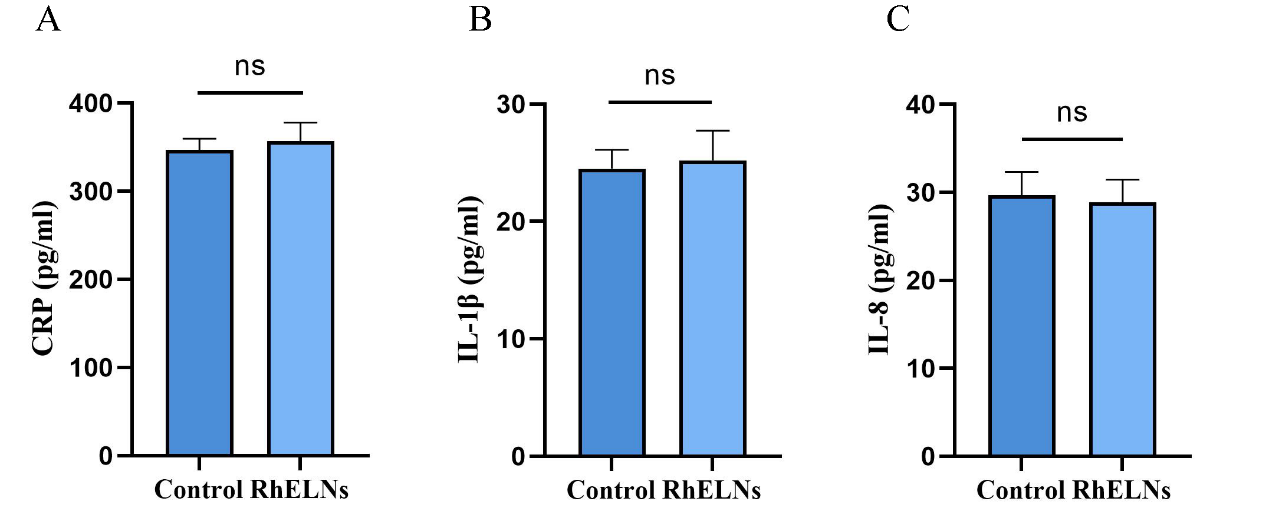


**FigureS7**. Effect of RhELNs on serum levels of CRP, IL-1β, and IL-8 in mice. *p < 0.05, **p < 0.01, ***p < 0.001. ns represents no statistical significance.


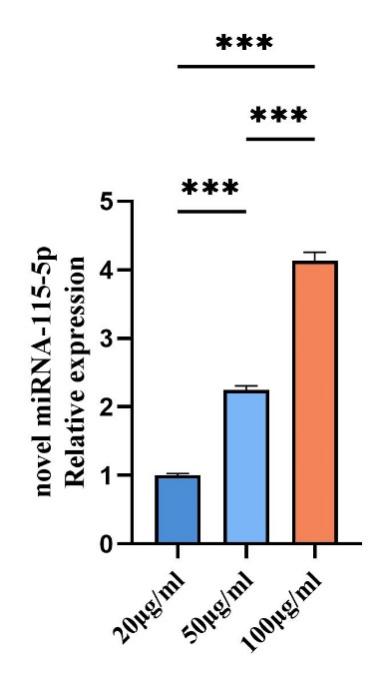


**FigureS8**. Relative expression changes of miRNA in cells after co-culture with HAECs at different concentrations of RhELNs. *p < 0.05, **p < 0.01, ***p < 0.001.


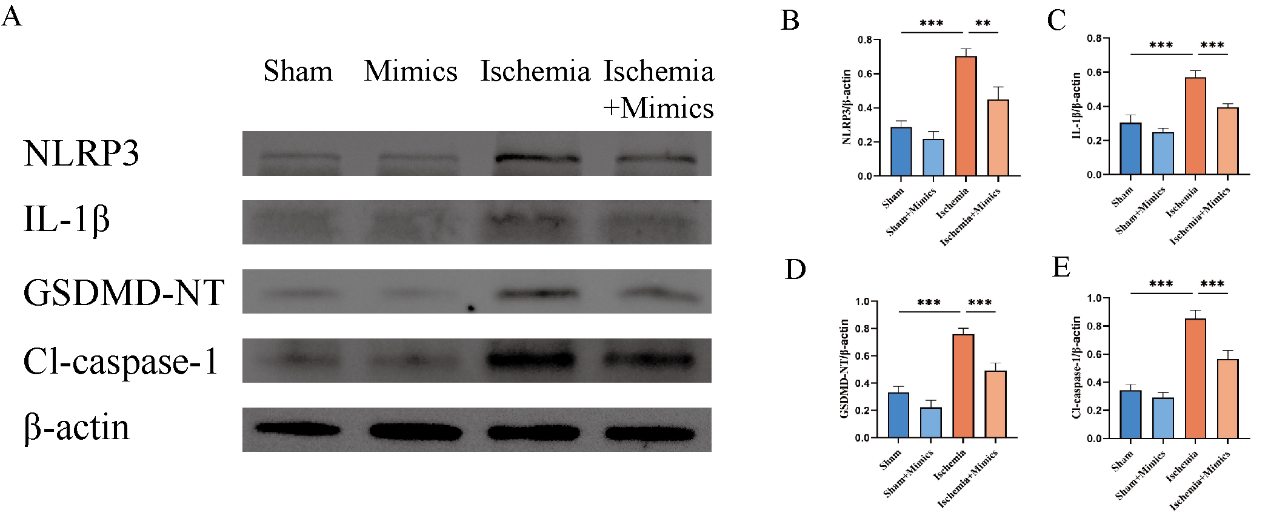


**FigureS9**. Western blot of NLRP3, IL-1β, GSDMD-NT, cleaved caspase-1, and β-actin expression in four mouse groups and quantitative analysis of relative protein expression. *p < 0.05, **p < 0.01, ***p < 0.001.


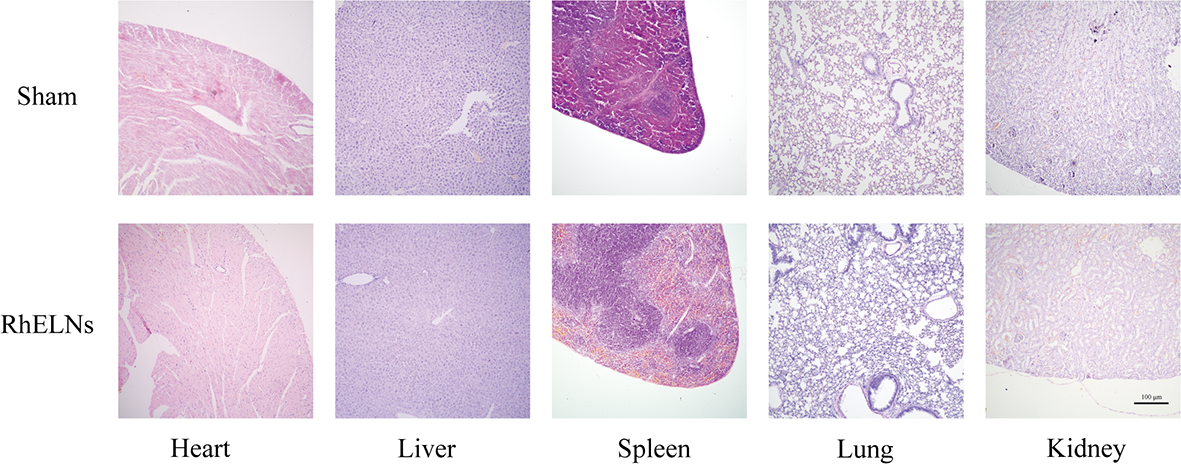


**FigureS10.** HE staining of major organs (heart, liver, spleen, lung, kidney) at 14 days post-RhELNs treatment.


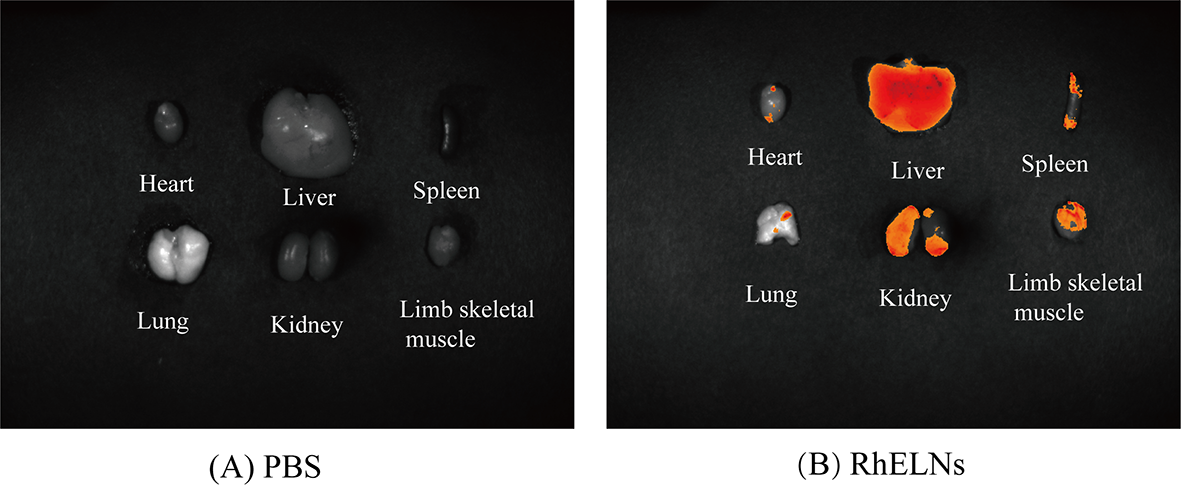


**FigureS11**. IVIS images of main organs and ischemic limb skeletal muscle of mice.

1. The IVIS image of major organs and ischemic limb skeletal muscle in mice at the same time point after PBS injection administered via the same injection method as the RhELNs treatment group. (B) The IVIS image of the main organs and ischemic limb skeletal muscles of the RhELNs treatment group mice (labeled dir) on day 3 post-surgery..
